# Overview of High-Performance Timing and Position-Sensitive MCP Detectors Utilizing Secondary Electron Emission for Mass Measurements of Exotic Nuclei at Nuclear Physics Facilities

**DOI:** 10.3390/s24227261

**Published:** 2024-11-13

**Authors:** Zhuang Ge

**Affiliations:** 1 Department of Physics, University of Jyväskylä, P.O. Box 35, FI-40014 Jyväskylä, Finland; zhuang.z.ge@jyu.fi; Tel.: +358-4578374864; 2 RIKEN Nishina Center, RIKEN, 2-1 Hirosawa, Wako, Saitama 351-0198, Japan; 3 GSI Helmholtzzentrum für Schwerionenforschung GmbH, 64291 Darmstadt, Germany

**Keywords:** mass spectrometry, B*ρ*-TOF, storage ring, Penning trap, MR-TOF, microchannel plates, detector, timing, position-sensitive

## Abstract

Timing and/or position-sensitive MCP detectors, which detect secondary electrons (SEs) emitted from a conversion foil during ion passage, are widely utilized in nuclear physics and nuclear astrophysics experiments. This review covers high-performance timing and/or position-sensitive MCP detectors that use SE emission for mass measurements of exotic nuclei at nuclear physics facilities, along with their applications in new measurement schemes. The design, principles, performance, and applications of these detectors with different arrangements of electromagnetic fields are summarized. To achieve high precision and accuracy in mass measurements of exotic nuclei using time-of-flight (TOF) and/or position (imaging) measurement methods, such as high-resolution beam-line magnetic-rigidity time-of-flight (Bρ-TOF) and in-ring isochronous mass spectrometry (IMS), foil-MCP detectors with high position and timing resolution have been introduced and simulated. Beyond TOF mass measurements, these new detector systems are also described for use in heavy ion beam trajectory monitoring and momentum measurements for both beam-line and in-ring applications. Additionally, the use of position-sensitive timing foil-MCP detectors for Penning trap mass spectrometers and multi-reflection time-of-flight (MR-TOF) mass spectrometers is proposed and discussed to improve efficiency and enhance precision.

## 1. Introduction

Mass is a fundamental property of the nucleus, which results from the intricate interaction between the strong, weak, and electromagnetic interactions that all nucleons experience [1]. Nuclear mass measurements have led to the discovery of new phenomena in nuclear physics, including shell structure, pairing correlations, decay, and reaction properties. Atomic masses can be used to deduce reaction *Q* values and separation energies. The boundaries of nuclear existence, known as the drip lines (*Sn* = 0 or *Sp* = 0), are identified based on the mass differences between neighboring nuclei. The masses of the involved nuclei also govern the final pathways of nucleosynthesis, such as the r-process and rp-process in the universe. Due to the importance of masses of nuclei in nuclear astrophysics and nuclear structure studies, there is a strong interest in fast, high-accuracy, and high-precision mass measurements for particularly exotic nuclides [2]. This has led to the development of a variety of techniques for mass measurement worldwide, including the following: the time-of-flight (TOF) measurements implemented at several facilities including the SPEG spectrometer at GANIL [3], the TOFI spectrometer/LANL [4], and the S800 Spectrograph/NSCL [5]; the Penning trap mass spectrometer ISOLTRAP/ISOLDE [6,7], JYFLTRAP/JYFL [8], SHIPTRAP/GSI [9], TRIGA-TRAP/MAINZ [10], CPT/CARIBU [11], LEBIT/MSU [12], and SMILETRAP/Stockholm [13]; Schottky mass spectroscopy (SMS) or the isochronous mass spectroscopy (IMS) method at the storage rings ESR/GSI [14,15,16] and CSRe/IMP [17]; multi-reflection time-of-flight mass spectrometer (MR-TOF MS) at ISOLDE [18], FRS Ion Catcher [19], and RIKEN [20]. Each advancement in building new mass spectrometers, whether through increasing resolving power or sensitivity, or both, has led to important new insights in physics.

This review article discusses the development of high-resolution timing and/or position-sensitive detectors that utilize SE emission for ion beam detection in mass measurements. These devices are based on the detection of SEs emitted following ion impact on a surface. Using a combination of magnetic and electric fields, the devices can achieve sub-nanosecond time resolution and sub-millimeter position accuracy for ion impacts, even at incidence rates exceeding $10^{6}$ ions/s. These advancements are expected to enhance the resolving power and sensitivity of current mass spectrometers, leading to significant breakthroughs in nuclear physics and nuclear astrophysics. The design, principle, performance, and application of timing and/or position measurements of all foil-MCP detectors being used for mass measurements, and other related applications both on the beam line and inside storage rings, are discussed. The detectors currently employed and novel ideas for future developments in accelerated ion beam facilities are summarized and envisioned. A possible new scheme of detector systems for low-energy or stopped ion beam facilities are proposed.

**Figure 1:**
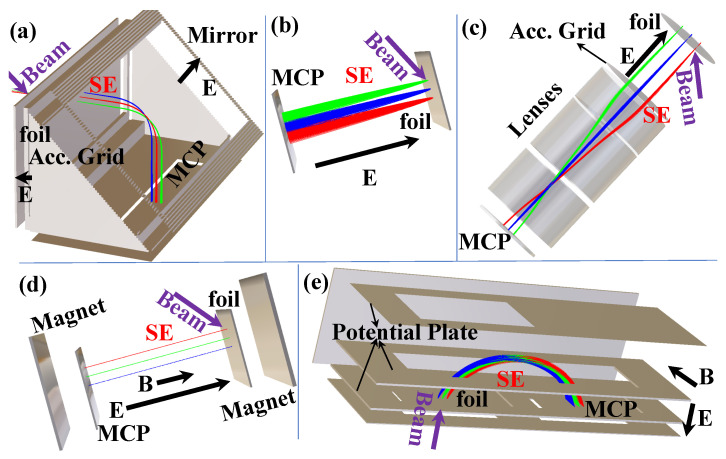
Schematic overview of the foil-MCP detectors: (**a**) Mirror-type electrostatic foil-MCP detector. (**b**) Direct projection electrostatic foil-MCP detector. (**c**) Electrostatic-lens foil-MCP detector. (**d**) Magnetic field and electrostatic field parallelly arranged foil-MCP detector. (**e**) Magnetic field and electrostatic field crossly arranged foil-MCP detector. The trajectory of the SEs are from simulations with SIMION.

In recent decades, there has been significant growth in the improvements of detection sensitivity and the precision of position and timing measurements, with an increasing focus on the overall performance of detectors. For mass measurements with accelerated ion beams with energies of a few hundred MeV/nucleon within 1 ms using magnetic-rigidity time-of-flight (Bρ-TOF) or isochronous mass spectrometry (IMS), the accelerated ion beam must not strike the Microchannel Plate (MCP) directly when using MCPs as transmission (beam) detectors. Instead, an electric and/or magnetic field is used to direct the secondary electrons (SEs) sputtered by the beam particle from an extremely thin conversion foil to the MCP. For time-of-flight measurements, the ultrafast multiplied electron signal from the MCP plate serves as the timing signal. The position of the beam particle can be determined by coupling the MCP to a position-sensitive anode. Foil-MCP detectors, which transport induced SEs from a thin foil towards an MCP detector with different arrangements of the electromagnetic field, as shown in Figure 1, are widely utilized in mass measurement experiments to deduce the timing and/or position information of heavy ions.

Foil-MCP detectors for timing determination (Figure 1e) have long been used in mass measurement experiments of exotic nuclei at three heavy-ion storage ring facilities: ESR/GSI [21,22,23], CSRe/IMP [24,25,26], and Rare-RI Ring/RIKEN [27,28,29,30,31,32]. These facilities have successfully performed precise IMS mass measurements using electrostatic and magnetic field crossly arranged (E× B) TOF detectors, either inside the ring for turn-by-turn revolution time measurement or outside for velocity/Bρ correction and revolution time deduction. In addition, the mirror-type timing foil-MCP detector (Figure 1a) has been used similarly outside the storage ring as the E×B TOF detector and in-ring for revolution time deduction at the Rare-RI Ring. Meanwhile, the position-sensitive foil-MCP detector with parallel electrostatic and magnetic fields (E‖B) used for Bρ-TOF mass measurements at NSCL/MSU [33] has demonstrated high performance and unique characteristics for position measurements, allowing for the deduction of the momentum/Bρ of exotic nuclei at a dispersive focal plane.

The previously described detectors were primarily optimized for timing measurements, sacrificing either timing information (ESR and CSRe) or good position resolution (NSCL), but with relatively small active detection areas. Recently, foil-MCP detectors with both high-precision timing and good spatial resolution for mass measurements and beam monitoring are being developed at existing and new-generation facilities such as RIBF/RIKEN [30,34,35,36], GSI-FAIR [37], and IMP-HIAF [38].

Foil-MCP detectors (Figure 1a–e) with position sensitivity play a crucial role not only in the tuning of the fragment separator but also in the particle identification of radioactive ion (RI) beams. They can be utilized to measure the positions and angles (or trajectories) of RI beams at the focal plane, enabling beam diagnostics and ion-optical tuning. In addition, position-sensitive detectors can be used for magnetic rigidity (Bρ) determination for the particle identification (PID) of RI beams, where trajectory reconstruction is employed to achieve high Bρ resolution and hence excellent PID power. Timing foil-MCP detectors are also desired for achieving the high-resolution PID of RI beams based on the magnetic-rigidity energy-loss time-of-flight (Bρ-ΔE-TOF) method. Based on the IMS technique, such as ESR/GSI [21,22], CSRe/IMP [24,25], and Rare-RI Ring/RIKEN [27,28,29], mass measurements could be performed with low energy loss of heavy RI beams based on the detectors. TOF measurements at two achromatic foci of the high-resolution beam line, along with additional Bρ measurements for TOF corrections, could help facilitate mass measurements via the well-developed Bρ-TOF method. It is also possible to combine Bρ-TOF mass measurements with the IMS mass measurements in a single experimental run, in which the in-ring revolution time is measured simultaneously with beam-line TOF and the Bρ of each RI [35,38]. This method was first realized at RIBF/RIKEN [35,38] and later proposed at IMP-HAIF [38].

To measure the most exotic areas of the chart of nuclides and to make effective use of precious beam time for efficient mass measurements, several types of foil-MCP detectors have been designed with the following characteristics: (a) very good timing resolution (<100 ps), (b) high position sensitivity and sub-millimeter resolution, low energy loss and small angular scattering of the heavy ions due to the use of a thin foil, (d) a large active area to cover a large beam size, (e) and high detection efficiency. High-resolution TOF is crucial for mass measurements via TOF methods, such as IMS in a storage ring or Bϱ-TOF with the beam line. The TOF information can also be used for velocity/Bϱ measurement for mass correction. High-resolution TOF measurements will also benefit high-resolution particle identification.

A precise position-sensitive Beam Profile Monitor (BPM) for each passing ion is crucial for measuring the momentum dispersion of radioactive ions (RIs). On the beam line, this BPM, combined with the high-precision TOF measurements and together with energy loss information in ΔE detectors like ionization chambers, enables the TOF−Bρ−ΔE method. This method allows for the deduction of the mass-to-charge ratio (A/q) and the proton number (*Z*) can be deduced, facilitating effective particle identification (PID) of the RIs due to the high performance of the detector. The accurate Bρ information of each ion can be used for mass correction in IMS (in ring or on the beam line) and directly utilized in Bϱ-TOF mass measurements.

For high precision and accuracy in mass measurements, the ideal case is a non-destructive detector. The energy loss and angular scattering should be minimized, which means using thinner foils with acceptable efficiency. A large active area to cover a large beam size is desired if the detector is located at a dispersive focus plane on the beam line. In consideration of the large dispersion and additional Betatron oscillation in a storage ring, a large active area is necessary for use in the ring. For efficient measurement, the detection efficiency should be as high as possible. The electromagnetic field added to the MCP detector should cause minimal disturbance to the isochronous ion-optics, and a correction of the in-ring electromagnetic field will be needed for multi-turn circulation of fully stripped heavy ions. Several types of detectors equipped with high-performance MCPs, which detect SEs induced by particles at a certain angle towards an MCP detector, can reconstruct the timing or position information of heavy ions. These detectors can satisfy the mentioned conditions above and serve as versatile instruments for mass measurements and beam diagnostics of exotic nuclei.

## 2. MCP Detectors for Mass Measurements of Exotic Nuclei

In studies requiring particle detection and identification for mass measurements, there is a significant demand for charged particle detectors with optimal position and timing resolution. Particle tracking in magnetic fields is often employed to filter reaction products in studies involving light particles at relativistic energies. Additionally, tracking more complex particles in large spectrographs and solenoids has been implemented in studies of heavy-ion-induced reactions. Detectors ideal for heavy-particle tracking are those that require minimal material for ions to pass through. While progress has been made with thin gas-filled detectors [39,40], such as Parallel Plate Avalanche Detector (PPAC) [41,42], Multiwire Drift Chamber (MWDC) [43], and Time Projection Chamber (TPC) [44], they need at least two strong foils to contain the active gas. This results in significant energy losses and scattering of the detected particles. Detectors using a single thin foil present an optimal solution. Very thin scintillators have been used as transmission detectors, but they require complex light collection mechanisms and must be thick enough to produce sufficient light [45]. In many cases, thicker foils have enhanced signal output, making these detectors essential for use with minimum ionizing particles [46,47,48]. This detection method has been successfully used in applications requiring good timing but have found limited use as position-sensitive detectors. An alternative approach involves detecting SEs emitted from a thin foil after an ion passes through it. These electrons are then multiplied in an MCP detector with a position-sensing anode. Since a large fraction of SE emission occurs at the surface, this method is, in principle, independent of foil thickness and should work with the thinnest possible foils.

MCP has been widely used as an amplifier in imaging and/or timing detectors at radioactive beam facilities due to its high gain, sub-nanosecond temporal response, low power consumption, stable operation in magnetic fields, sensitivity to a single electron, and compact size [49]. Foil-MCP detectors, which transport induced SEs from a thin foil towards the MCP surface using different arrangements of the electromagnetic field to deduce the timing and/or position information of heavy ions, are widely utilized in nuclear physics experiments. All the above-described detectors were mostly optimized for timing measurements at the cost of only timing information (ESR/GSI [21,22], CSRe/IMP [24,25], and Rare-RI Ring/RIKEN [27,29]) or good position resolution but with relatively worse timing resolution (NSCL/MSU [5]), and relatively small detection active areas. Electrostatic mirror-type foil-MCP detectors with good timing performance and medium spatial resolution for mass identification studies and beam monitoring have also been developed in many laboratories worldwide [34,50,51,52,53,54,55]. Compact beam timing E×B MCP detectors have also been used to measure the TOF of beam particles and reaction products in nuclear reaction studies [56,57].

MCP detectors with timing anodes have been commonly employed in the traditional time-of-flight ion-cyclotron-resonance (TOF-ICR) method [8,58,59,60] for Penning trap mass spectrometers. The cutting-edge phase-imaging ion cyclotron resonance (PI-ICR) technique [60,61], utilizing a position-sensitive MCP detector, offers approximately 40 times higher resolving power compared to the TOF-ICR method and has become the predominant technique for mass measurements in Penning trap mass spectrometers. With a typical efficiency of 15–35% [61], the PI-ICR technique, equipped with an additional amplification of the incident ion by the configuration of Figure 1b, will highly enhance the efficiency. Further studies of the yield of the SEs from low-energy incident ions of a few tens of keV need to be carried out. Foil-MCP detectors of this type are expected to achieve a detection efficiency of 90–100% with a compact structure. If implemented, this method could result in achieving the same precision approximately 10–40 times faster for Penning trap mass spectrometers. This will allow the mass measurements of the nuclide chart to cover regions with one or two more neutron-rich and proton-rich areas. The MR-TOF mass spectrometer typically incorporates a timing detector for mass measurements [18,19,20]. Integrating a position-sensitive MCP detector, which offers both timing and position sensitivity, can possibly significantly enhance the resolving power of these mass spectrometers. This improvement is due to the strong correlation between the isochronous TOF of ions and their position information. Correcting the TOF spectrum based on the two-dimensional positions of the ions can maintain and enhance the resolving power. Furthermore, aligning the beam within the MR-TOF mass spectrometer using the measured position information can enhance transmission efficiency and maintain high resolving power at the start of the mass measurements. In addition, α-TOF and β-TOF detectors [62,63] based on these detector types coupled to an integrated Si detector can be designed for correlated measurements of atomic masses and decay properties of radioactive isotopes using MR-TOF or Penning trap mass spectrometers.

### 2.1. Detector Design and Principle of Operation

Detectors that require ions to pass through a single foil to determine the time and position of the ion have been widely used. Figure 1 illustrates the schematic layout of such detectors, designed to capture the timing and position of ion impact on a thin foil. SEs are emitted from the foil at the ion impact location and are accelerated toward a fast electron multiplier, such as an MCP. This setup, and similar ones, provide an accurate timestamp for the ion’s impact on the foil and have been successfully used in fast-timing applications [21,22,24,25,27,29,50,51]. Electron ejection from the foil can be confined to the position where the ions hit the foil. Therefore, if the anode of the MCP detector is replaced by a position-sensing anode, this detector could also record the position of electron impact on the detector surface, indirectly providing information on the ion’s impact position on the foil. There are several methods to achieve position sensitivity with an MCP detector [64], including multi-strip anode [65], helical delay line [66,67], cross-strip anode [68], induced signal [69,70], resistive anode [71,72,73], and Timepix CMOS readout [74]. Position resolution for several direct SE projection electrostatic foil-MCP detectors (Figure 1b), where foil-to-MCP distances are at least a few centimeters, is no better than 2 mm (FWHM), as reported in [75]. Ref. [76], which analyzes the performance of this type of detector, identifies electron transport from the foil to the detector as the main obstacle to achieving sub-millimeter resolution of the heavy-ion impact position on the foil. The primary cause of deviation from a straight path for the SE trajectory is their initial lateral velocity for the type of detector in Figure 1b. This lateral component’s impact can be minimized by rapidly accelerating the SEs toward the detector [75]. Mirror-type detectors (Figure 1a) have been reported to achieve approximately 1 mm (FWHM) resolution power and a timing resolving power of a few tens of ps [50,51] due to the mirror structure design. It is suggested in Ref. [77] that optimal preservation of the information on the points of origin during electron transport from foil to MCP detector surface will require a combination of magnetic containment and electrostatic acceleration. It has also been recognized that a magnetic field parallel to the desired propagation direction will cause the electron to move in a tight spiral from the foil to the MCP detector surface [77]. In Ref. [77], low magnetic fields were applied, and accurate localization was based on the number of full turns executed by the electrons spiraling along the central trajectory. Applying a strong magnetic field in the direction of the desired electron motion should improve the correspondence between the electron’s generation location and its arrival at the detector. The design depicted in Figure 1d, the so-called beam parallelizer, is used to collect electrons of a few eV energy diverging from an SE source and turn them into a parallel beam. A strong, but not necessarily uniform, magnetic field and minimal electron acceleration are employed. Sufficient acceleration is performed with an electrostatic field to keep the time spread in electron transit from foil to MCP detectors low. Electron motion under the combination of small electrostatic acceleration and an inhomogeneous magnetic field [77] is described in Appendix A.2. This type of detector achieved a two-dimensional position resolving power of sub-millimeter resolution [5,78] (approximately 0.5 mm resolution power in FWHM has been achieved with a timing resolution of a few hundreds of ps). A serious limitation of this type of detector is the large space occupied by this detector in a parallel arrangement of the electrostatic and magnetic fields, making their use prohibitive in many experiments. E×B detectors, known for their compact design and minimal material introduction into the beam path, normally serve as beam timing detectors [21,22,25,27,29,79,80,81,82], and have achieved the best resolving power of 20–40 ps. This type of detector has been utilized to measure the time of flight of beam particles and reaction products in nuclear reaction studies [56,57]. To achieve one-dimensional position sensitivity in the MCP of an E×B detector, a multi-strip anode with delay line readout was employed in [83], which is particularly appealing due to its simplicity and low cost. The spatial resolution can reach values better than 0.5 mm (FWHM) [79,83]. A recent proposal to use it as a position-sensitive timing detector in ring was reported in [37].

All possible high-performance timing and/or position-sensitive foil-MCP detectors that could be used for high-precision mass measurements of exotic nuclei are summarized as shown in Figure 1:

1. Figure 1a shows the mirror-type electrostatic foil-MCP detector. Typically, a timing resolving power of around 40 ps (in σ), as reported in [30,84], can be reached using a timing anode coupled to an MCP. A timing and position resolving power of around 50 ps (in σ) and ∼1 mm (in σ) can be achieved using a delay-line anode for this type of foil-MCP detector [36,84]. The timing resolution will highly depend on the size of the detector.
2. Figure 1b shows the direct SE projection electrostatic foil-MCP detector. A good timing resolving power of a few tens of ps can be achieved, but only when the transportation of SEs to the MCP is within a short distance and fast enough to reach a sub-mm position resolving power, typically more than 2 mm (FWHM) [75], to achieve a relatively large active area.
3. Figure 1c shows the electrostatic-lens foil-MCP detector. It has a sub-mm position resolution and a timing resolving power of a few tens of ps.
4. Figure 1d shows the magnetic field and electrostatic field parallelly arranged foil-MCP detector. It has good position resolving power (sub-mm) but a timing resolving power of a few hundreds of ps.
5. Figure 1e illustrates the magnetic field and electrostatic field crossly arranged foil-MCP detector. It has good position resolving power, reaching around 30 ps, and one-dimensional position sensitivity with sub-mm position resolving power.

The trajectories of the SEs when using these detectors, as simulated by SIMION [85], are schematically illustrated in Figure 1.

#### 2.1.1. Conversion Foils and Secondary Electron Yield

When charged particles pass through a thin solid material, SEs will release. These SEs typically have an energy of a few electron volts (eV) for the primary components [86,87]. Under high voltage differences, these SEs can be accelerated and collected to provide information related to their creation location and time. Typically, carbon foils with thicknesses ranging from 10 to 100 μg/cm^2^ and Mylar foils with evaporated aluminum or gold layers are used, considering factors like straggling, efficiency, and fragility. Thin foils are ideal for minimizing straggling, but they are more prone to structural failure. Mylar is stronger than carbon, and the high atomic number (Z) of aluminum/gold increases the MCP signal amplitude, enhancing detection efficiency. However, Mylar and aluminum/gold foils cause excessive straggling. Foils can be produced using various techniques, including sputter deposition, pulsed laser ablation, ion beam deposition, and plasma-enhanced chemical vapor deposition. Ref. [88] describe a complex process for creating carbon foils of 2 μg/cm^2^, starting with an evaporation step followed by the addition of a polyvinyl formal layer, which is later removed to obtain the self-supporting carbon foil. Ref. [89] emphasizes the benefits of the filtered cathodic vacuum arc method for synthesizing highly transparent and self-supporting films. Ref. [90] describes the preparation of foils as thin as 0.6 μg/cm^2^ using glow discharge sputtering of graphite in a low-density krypton plasma. However, thinner foils are not always better for the application discussed in this paper. While they minimize energy loss and straggling of the heavy-ion beam, factors like homogeneity and robustness are also crucial in the design.

Ions passing through the foil generate SEs primarily through inelastic atomic collisions with electrons within the detector foil. The SE emission process involves three main steps [91]: (i) generation of ionized atoms and free electrons within the solid, (ii) diffusion of the electrons to the target surface, including cascade multiplication, and (iii) reduction of the ions’ kinetic energy by the work function at the surface (few eVs for solid carbon) [92]. The amount of introduced SE counts $n_{SE}^{t}$ within the material at depth *x* is directly proportional to its energy loss per unit path length dE/dx [μg/cm^2^/keV] [87,91,93,94,95,96,97]: $n_{SE}^{t}=\Lambda \frac{dE}{\rho dx}$, where ρ is the density of the foil and the value of constant Λ depends on the material of the foil. The secondary electron yield per incoming particle has been well studied [87,93,98]. For example, Λ is 7 μg/cm^2^/keV for carbon [87], 13 μg/cm^2^/keV for aluminum, and 60 μg/cm^2^/keV for gold [99] targets. Despite the systematic deviations related to the dependencies on projectile and target proton numbers discussed in [87], the assumption of a general proportionality between SE yields and the electronic energy loss of the projectiles is impressively demonstrated in Figure 12 of Ref. [87]. This figure illustrates the total SE yield from carbon foils as a function of the electronic energy loss. When a fast ion travels through a solid, it loses kinetic energy via distant or close collisions with electrons, producing slow secondary electrons (SSEs) and fast delta (δ) electrons, respectively. Both the primary ion and δ electrons contribute to SE cascade multiplication. Due to their distinct generation mechanisms, SSEs exhibit isotropic angular distributions, while δ electrons display forward-directed angular distributions [93,94]. Semiempirical equations for the yields of the secondary electrons per ion in forward and backward directions can be written as [93,98]:

(1) $$n^{F}\left(d\right)=\Lambda \left(\frac{dE}{dx}\right)\left[1-\beta_{S}e^{-d/(\lambda_{S})}-\beta_{\delta }e^{-d/(\lambda_{\delta })}\right],$$

and

(2) $$n^{B}\left(d\right)=\Lambda \left(\frac{dE}{dx}\right)\beta_{S}\left[1-e^{-d/(\lambda_{S})}\right],$$

where Λ is a constant that mainly depends on the target material. Partition factor $\beta_{S}$ ($\beta_{\delta }=1-\beta_{S}$) is a partition factor of ion energy loss for SSE (δ electron) and about 0.2 for protons and helium, and for heavier ions, $\beta_{\delta }$ increases up to 0.7 [98]. The total electron yield of the slow secondary electrons per ion is the sum of the yields in forward and backward directions: $n_{SE}^{t}\left(d\right)=n_{SE}^{F}\left(d\right)+n_{SE}^{B}\left(d\right)$. $\lambda_{S}$ and $\lambda_{\delta }$ are the SSE and δ electron transport lengths, which are equivalent to the mean free path in the target. In the case of heavy ion ^100^*Sn*^50+^ (170 MeV/nucleon) passing through the carbon foil with a thickness of ∼40 μg/cm^2^, ∼50 secondary electrons will be emitted in the forward direction and ∼20 electrons in the case of α particle (4–6 MeV) [93,95]. From 2 μm Mylar foil coated with aluminum (100 nm), the secondary electron yield is about 60 for the heavy ion ^100^*Sn*^50+^ with an energy of 170 MeV/nucleon. To improve the yield of SEs, gold (Λ∼64 μg/cm^2^/keV) [99] and CsI (Λ∼82 μg/cm^2^/keV) [21] will be effective because more electrons are expected due to the large Λ values. The detection efficiency of the MCP is influenced by the energy of the SEs and reaches a plateau for electron impact energies above 500 eV [79,100]. Before reaching the MCP, the electron impact energy can be accelerated to exceed 500 eV. With an open area ratio of typically ≈60% for the MCP device and a designed transport efficiency of over 90% for various foil-MCP detectors, the detection efficiency for more than one electron is estimated to be over 80%, and nearly 100% for more than two secondary electrons, for both forward and backward directions from the foil.

#### 2.1.2. Simulation

To demonstrate the design and performance of these SE detection detectors, simulation of the SE transport from the conversion foil was conducted using the SIMION software package [85]. In these simulations, SE energies were assigned a range of 0–20 eV, with a mean value of approximately 2 eV, representing the primary electrons emitted from the foil with a percentage of 85% [86,87,101]. The emission angles ranged from −90 to +90 degrees relative to the foil surface, and SEs emitted from both the forward and backward sides of the foil are directed to the MCP surface. Each parameter of the uniformly distributed electrons was generated using a random generator in SIMION [85].

The real electric field usually contains disturbances compared to the perfectly designed electric field. Because of that, the timing resolution and spatial focusing can be different from that of the calculated ideal case. However, the analytical solution (simulation) can be useful to define the direction of the investigations. In order to determine the timing and position resolutions, SEs were assigned to groups and start from the same point of the foil. As shown in Figure 1a–e, a three-point imaging of grouped SEs from the foil onto the MCP surface was studied. Different high-voltage (HV) supplies were added to the potential plates or grids and varied during the simulation for different settings. In addition, the magnetic field, when present, was varied and investigated. The TOF distribution, and two-dimensional position distributions of initial SEs for each group from the foil reflected onto the MCP were analyzed. The simulation results are briefly discussed in the following sections.

In the following Section 2.2, Section 2.3, Section 2.4 and Section 2.5, advanced detector designs with both timing and position sensitivity will be discussed in detail. To achieve both high timing and position sensitivity, some of these designs used a segmented structure, taking advantage of both forward and backward SEs and employing two of the basic detector types, as shown in Figure 1.

### 2.2. Electrostatic Mirror Detector

**Figure 2:**
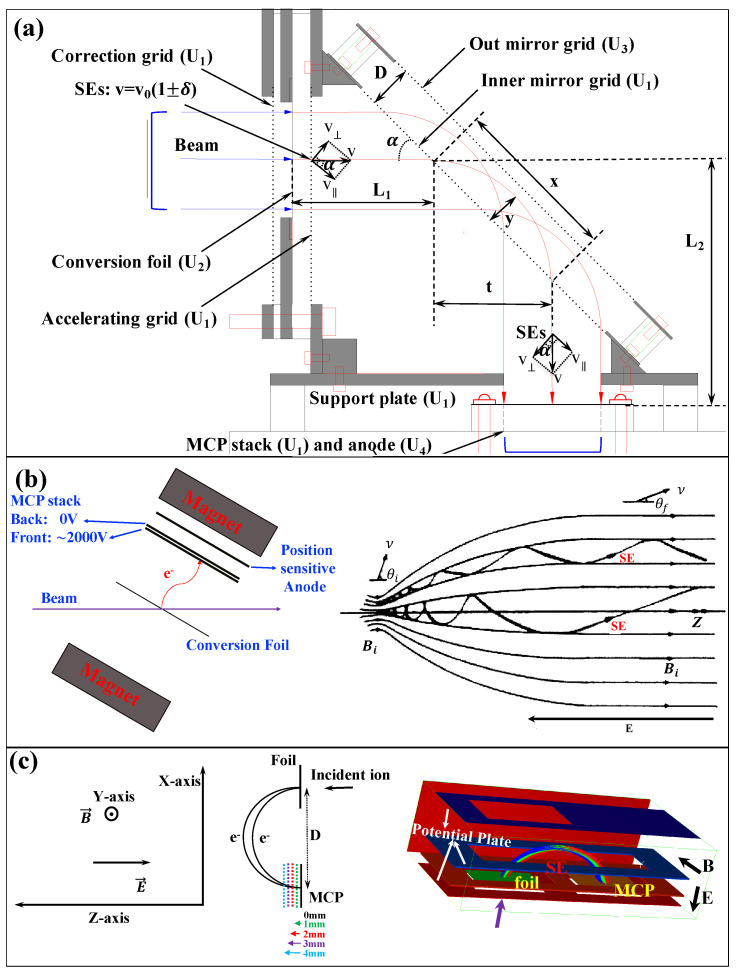
Schematic diagram of the working principle of foil-MCP detectors. (**a**) Schematic view of the trajectories of SEs from the conversion foil to the MCP detector for the electrostatic mirror detector [36]. (**b**) Schematic diagram illustrating the principle of a B‖E-MCP detector. The trajectories of the SEs in a magnetic field that changes gradually from a strong field to a weaker uniform field is modified from [102]. (**c**) This setup represents a cross-type B×E-MCP detector. Heavy ions travel along the positive z-axis, the electric field is oriented along the negative z-axis, and the magnetic field is reversed along the positive y-axis [103].

The electrostatic mirror MCP detector system, as illustrated schematically in Figure 2a, typically comprises a conversion foil, an accelerating grid, an electrostatic mirror, an equipotential housing, and an MCP assembly. When ions penetrate the conversion foil, SEs are produced and then accelerated by the accelerating grid wires. After acceleration, the SEs enter the detector interior, pass through a field-free region, and are then bent by the electrostatic mirror harp wires. Finally, the SEs drift freely to another field-free region, reach the MCP front surface, and are detected by the coupled anode. These processes are schematically shown in Figure 2a; thus, the detector can be divided into three main functional parts:

1. SE generation and acceleration: front wall with a conversion foil at a potential $U_{2}$, and an accelerating plate with grids at potential $U_{1}$.
2. SE reflection: an electrostatic mirror plate at $U_{3}$ with grids as the back wall to reflect SEs.
3. SE field-free region and SE detection: the bottom plate ($U_{1}$) with a circular or rectangular hole, two side walls ($U_{1}$) to maintain equal potential inside and serve for fixing, and a chevron-type MCP (front surface at $U_{1}$) with a delay-line anode ($U_{4}$) to detector SEs.

Additionally, an additional aluminum plate (at $U_{1}$) with grids is placed in front of the foil to balance the force on the foil and prevent deformation or breakage due to electrostatic force. The construction materials of the detector were selected to meet both the high voltage requirements (dielectric strength) and vacuum conditions (material degassing) inside the detector. The conductive plates are made of aluminum and all the grids consist of gold-plated tungsten (W+Au) (40 μm in diameter). The detector can operate solely with an electrostatic field [34,36] to avoid disturbing the isochronous field in the storage ring, Rare-RI Ring, which consists of 24 dipoles [35,104]. Carbon foil with 10–60 μg/cm^2^ thicknesses or Mylar foil coated with aluminum with thicknesses of 2–4 μm is used as the conversion foil for SEs emission. When the detector is used for timing only, the MCP is coupled with a timing anode [30]. If a two-dimensional position-sensitive delay-line anode is mounted below the MCP, it can be used for both timing and position measurements simultaneously [36]. To attain high timing and spatial resolution, it is crucial to carefully calculate the motion of SEs within the detector and their interaction with the potentials applied to the detector plates. A detailed description of the electromagnetic motion of SEs can be found in Appendix A.1. Furthermore, a simulation study is required to determine the optimal design based on the specific requirements.

**Figure 3:**
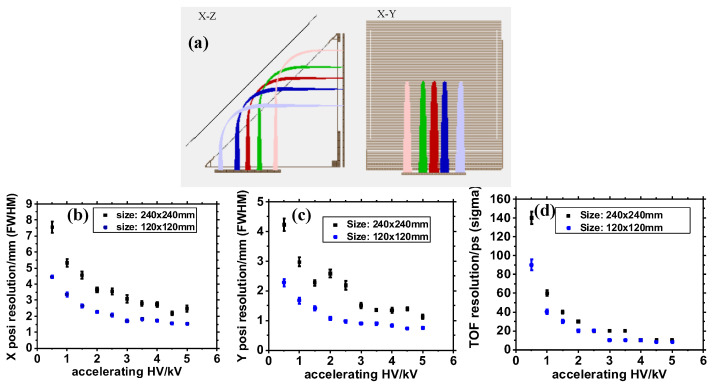
(**a**) Five-point imaging of SEs from the foil onto the MCP surface in the X-Z and X-Y views during the simulation. The comparison of X-coordinate position (**b**), Y-coordinate position (**c**), and timing (**d**) resolutions for detectors with different dimensions (120 mm × 120 mm and 240 mm × 240 mm for the triangular structure). The HV settings for the different plates were identical, and the accelerating HV values were all negative in the simulation [36].

To optimize the timing and position resolutions of this type of detector, a simulation was conducted. SEs were grouped according to the distribution described in Section 2.1.2 and started from five points on the foil. The simulation included a five-point imaging of these grouped SEs onto the MCP surface, viewed in the X-Z plane (left panel of Figure 3a) and the X-Y plane (right panel of Figure 3a). Different high-voltage (HV) supplies were applied to the potential plates or grids and varied for different settings during the simulation. The TOF distribution and two-dimensional position distributions of initial SEs from the foil, reflected onto the MCP, were fitted with a Gaussian function. The peak width was characterized by the Full Width at Half Maximum (FWHM), defined as 2.355σ, where σ is the standard deviation of the Gaussian distribution.

A comparison of timing and position resolutions of different dimensions (120 mm × 120 mm and 240 mm × 240 mm for the triangular structure) was simulated and the results are demonstrated in Figure 3. The results clearly indicate that smaller-sized detectors offered better position and timing resolutions when the HV supplies were set identically. This improvement was due to the reduced total TOF and path length of SEs in a more compact structure, which minimizes the influence of the initial energy and angular distribution of SEs from the foil. The trends in timing and position resolutions for the mirror detector, depicted in Figure 3, show that increasing the accelerating HV enhanced these resolutions until they finally became nearly saturated at a plateau. An intrinsic timing resolution typically better than 20 ps and two-dimensional position resolutions of ∼1 mm were achieved.

### 2.3. Electrostatic-Lens Position-Sensitive TOF MCP Detector

**Figure 4:**
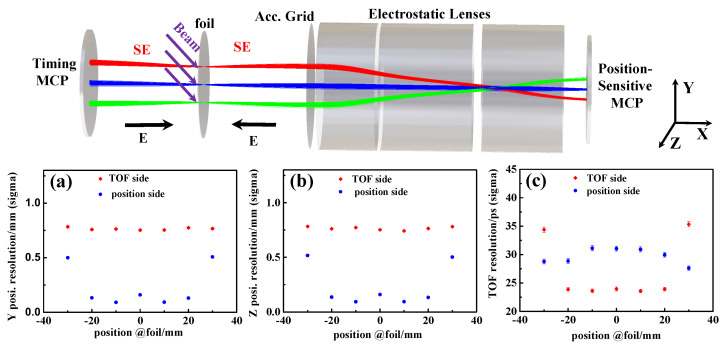
(Upper panel) Simulation of SE trajectory in an electrostatic-lens MCP detector. The lower panel illustrates the comparison of the Y-direction (**a**), Z-direction (**b**) position resolution, and timing (**c**) resolution as a function of the position at the foil for the timing side (depicted as TOF side in the legend) and position-sensitive side (depicted as position side in the legend), respectively [38].

The 3D structure of the designed electrostatic-lens position-sensitive TOF MCP detector is shown in Figure 4, integrating two segmented parts as depicted in Figure 1b,c. It comprises a conversion foil, an accelerating grid, a triplet electrostatic lenses, two MCPs with a timing anode made of metal at the rear side, and a two-dimensional position-sensitive delay-line anode [105] at the forward side, respectively. The detector configuration features a straight structure, with the conversion foil tilted at an angle of 30^∘^ relative to the heavy-ion beam axis. A self-sustained carbon foil with a thickness of 5–20 μg/cm^2^ (25–100 nm) or 140–280 μg/cm^2^ (1–2 μm) Mylar foil coated with aluminum with a thickness of 27 μg/cm^2^ (100 nm) can be used as the conversion foil for SE emissions. The accelerating grid which possesses crossed wires with 1 mm pitch in both directions can be made of gold-plated tungsten (W+Au) with a diameter of 40 μm.

As illustrated in Figure 4, the detector comprises two functional areas: one in the backward direction for timing determination and another in the forward direction for position measurement. The backward SEs, induced from the foil upon impact by a heavy ion, are directly accelerated to the MCP via an accelerating potential of −4800 Volts (V) between the MCP (biased at 2400 V) and the foil (biased at −2400 V), allowing the timing information of the ion’s impact to be recorded. Meanwhile, the forward SEs emitted by the same ion are accelerated by the accelerating grid (biased at −2000 V) and focused onto the forward MCP (biased at 2400 V), which is coupled with a position-sensitive anode to reproduce the position information of the ion on its impact. The electrostatic lenses help constrain the position dispersion of the SEs and focus them onto the MCP (biased at 2400 V) surface to preserve their position information. The high-voltage supply values for each electrode of the triplet-lens system are specified [38]. A key feature of this MCP detector is its minimal beam perturbation compared to the mirror-type MCP detector, as there are no wires along the passage of RI beams in the MCP detector.

To enhance the design and performance of the MCP detector, a simulation of the transport of secondary electrons (SEs) induced from the conversion foil was conducted using SIMION [85]. As illustrated in Figure 4, the resulting position and timing resolutions are shown for both sides. The Y- and Z-coordinate position resolutions of the MCP detector at both the timing-sensitive and resolution-sensitive sides, as a function of the corresponding positions on the foil, are depicted in Figure 4a,b. In Figure 4c, the timing resolutions of the position-sensitive side and the dedicated timing side, as a function of the corresponding positions on the foil, are presented. The target position resolution and timing resolution for this type of detector are ≤1 mm (in σ for two dimensions) and 20–40 ps (in σ) with an effective area of Φ60 mm. Future designs aim to expand the effective area of this type of detector to as large as Φ100 mm.

### 2.4. E×B Position-Sensitive Timing MCP Detector

**Figure 5:**
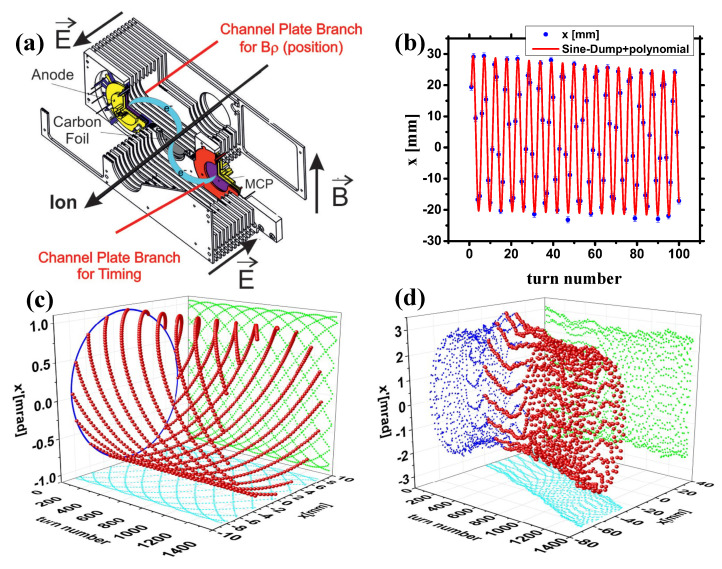
(**a**) The B×E foil-MCP detector designed to provide both timing and one-dimensional positional sensitivity [37]. (**b**) The positional data (*x*/mm) for ions during each revolution are recorded by a position-sensitive detector that experiences energy loss (using a foil-MCP detector) within the storage ring [103]. The error bars for each data point (in bule) in the simulation account for a resolving power of 1 mm (σ) for the foil-MCP detector. A function: $x=x_{0}+a\cdot exp\left(-b\cdot T\right)\cdot sin\left(2\pi \cdot \left(T-T_{0}\right)/\omega \right)+c\cdot T$, is used to fit (in red) the betatron oscillation (see Section 5.3 for details). (**c**) The positional and angular data (*x*,$x^{\prime }$) for ions during each revolution are captured by a position-sensitive detector that functions without any degradation (for example, using a Schottky pickup as the probe) within a storage ring. (**d**) The positional and angular data (*x*,$x^{\prime }$) for ions per revolution obtained by a position-sensitive detector that experiences energy loss (utilizing a foil-MCP detector) within the storage ring [103]. The ions being simulated in this scenario are ^38^*K*^19+^ with an energy level of approximately 200 MeV/nucleon. The simulation is based on the COSY [106] and MOCADI [107] software packages, developed at MSU and GSI.

The B×E-MCP detectors with uniform electric and magnetic field crossly arranged are routinely used in the mass measurement experiments of heavy-ion storage rings at GSI, IMP, and RIKEN. Figure 2c and Figure 5a illustrate the working principle of the B×E detector. When an ion passes through, secondary electrons (SEs) are emitted from both surfaces of the foil. The crossed static electric and magnetic fields cause the SEs to follow a cycloid motion, transporting the SEs isochronously to one or two MCP detectors positioned in the forward and backward directions of the ion beam. Despite the SEs being emitted with a wide angular spread, the acceleration with the electric field reduces their angular dispersion. This electromagnetic field ensures a isochronous electron transport. The SEs of three ion beam spots are focused in one dimension at the MCP surface, with their simulated trajectories shown in Figure 1e. In a uniform electromagnetic field, the center-to-center distance *D* from the foil to the MCP detector (Figure 3c) is determined by the field’s strength. The electromagnetic motion of secondary electrons is detailed in Appendix A.2.

The detector model shown in Figure 1e is a large-area electromagnetic field B×E-MCP detector designed for the next-generation heavy-ion accelerator HIAF with an effective area of 50 mm × 200 mm. As shown in Figure 3c and discussed in Appendix A.2, only when the displacement difference between the MCP surface and the surface of the conversion film is >0 mm and the electric field strength is relatively large, can the SEs emitted from the conversion film reach the MCP surface smoothly. Table 1 gives the two-dimensional position (X and Y) and timing resolution of the detector by simulation with SIMION [85]. The displacement difference between the MCP surface and the conversion film surface is 0–4 mm. The magnetic induction intensity of the magnetic field set in the simulation is B = 85 gauss, and the electric field strength E = 200 V/mm. This detector can be designed as a one-dimensional position-sensitive TOF detector, at which the timing information of heavy ions is obtained by measuring the forward SEs by the time-sensitive MCP detector, and the position information of the heavy ions are obtained by measuring the forward SEs by the position-sensitive MCP detector. This type of development is proposed at ESR/GSI [37]. The position resolution of the target is better than 0.5 mm (σ, one dimension) and the time resolution achieved is better than 50 ps (σ). The newly designed MCP detector for HIAF simulates an intrinsic timing resolution of better than 20 ps (σ) and an intrinsic position resolution of 0.15 mm (FWHM, 1 dimension), as shown in Table 1.

### 2.5. E‖B Position-Sensitive Timing MCP Detector

A position-sensitive timing foil-MCP detector with parallel electrostatic and magnetic fields (beam parallelizer) can be used to collect electrons with a few eV of energy diverging from an SE source and convert them into a parallel beam. This is achieved by applying a strong, though not necessarily uniform, magnetic field and minimizing electron acceleration. This limited acceleration helps reduce the time spread in electron transit from the foil to the MCP detectors. The motion of SEs under small electrostatic acceleration and an inhomogeneous magnetic field is detailed in Appendix A.2.1. In this setup, lateral momentum is gradually converted to longitudinal momentum. Provided the magnetic field is strong and the linear acceleration due to the electric field is moderate, the electrons will spiral along the magnetic field lines. This concept relies on adiabatic invariance [76,102]. Electrons emitted from the foil are accelerated towards the MCP and generally move along the magnetic field. Electrons with an initial transverse momentum will spiral along the magnetic field lines. If the magnetic field is constant, this spiral motion results in periodic circular motion in a plane perpendicular to the magnetic field. The SEs spiral around the diverging magnetic field lines, and the transverse component of their velocity gradually decreases as the magnetic field strength diminishes from its initial value at the foil. As mentioned earlier, adiabaticity (the conservation of the action integral) can be expressed in terms of the constancy of the flux linked by the orbit of SEs. This leads to an expression where the ratio representing the change in orbit radius defines the lateral image (de)magnification, *M*, of the orbital motion. See Appendix A.2.1 for the details of the principle.

**Figure 6:**
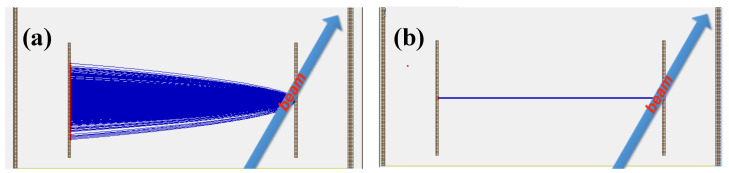
Simulation of SE trajectories in the absence of magnetic field (**a**) and magnetic field (**b**) [85]. The SEs of the detector are emitted from the conversion film of the detector. The implementation of an additional magnetic field can significantly improve the confinement of the ion’s position.

Figure 6 is the simulated adiabatic process without a magnetic field confining the lateral motion of electrons (a) and the applied magnetic field confining the lateral motion of electrons (b). After adding a magnetic field to the detector, the lateral motion of the electrons is strongly confined within a small offset range, and the constraint of the magnetic field on the lateral motion of the electrons greatly improves the position resolution of the detector.

This type of detector has good position resolution, straightforward construction, effortless maintenance, and low requirements for magnets that supply magnetic fields. It stands out as a superior alternative for position measurement within the beam lines HFRS and storage ring SRing of next-generation heavy-ion devices HIAF. Table 2 gives the two-dimensional position (X and Y) and timing resolution of the simulated MCP detectors arranged in parallel with the electromagnetic fields when the electric and magnetic field strengths change. The detector is designed to achieve a positional resolution of <0.5 mm (FWHM, two dimensions), and a timing resolution better than 300 ps (σ).

## 3. Calibration of the Position-Sensitive Anode of an MCP Detector

**Figure 7:**
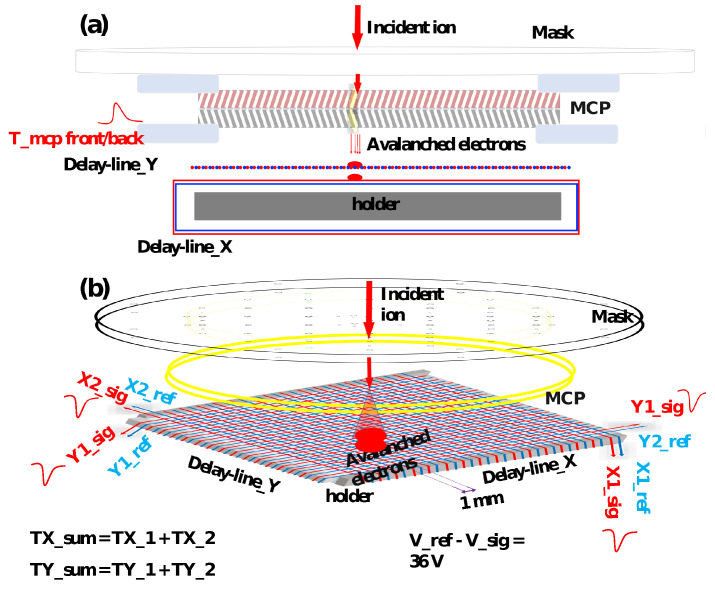
(**a**) shows the schematic cross-sectional view of the setup for the calibration of the DLD system. (**b**) indicates the 3D imaging principle of the calibration setup [36].

Here, we use the helical delay-line MCP detector as an example to discuss the calibration of the anode of a position-sensitive MCP detector. The helical delay-line anode of the delay-line MCP detector (DLD) [105], as shown in Figure 7, consists of a holder and two coils for the X and Y directions. Each coil (dual delay line) with two wires is wound in parallel with a pitch of 1 mm around the holder. One wire acts as a reference wire (Reference) and the other as a charge collection wire (Signal). The holder has a metal core with four checkered ceramic insulators at the edges. The delay lines are wound around these insulators, the first in a direction with a smaller circumference (X), and the second in a direction perpendicular to the first with a larger circumference (Y). The Signal wire is at a more positive potential (+36 V) than the Reference wire, making it more attractive to the electrons coming from the MCP back plate and producing a larger signal. When an electron cloud propagates along a collecting wire, a current is induced only in the neighboring Reference wire, which can then be registered. The anode holder acts as a reflecting plate, ensuring that nearly all electrons from the charge cloud are collected by the Signal wire. The single pitch propagation time (for 1 mm) on the delay line is approximately 1.24 ns for the DLD. Therefore, the correspondence between 1 mm position distance and relative time delay in the two-dimensional image is twice this value: approximately 2.48 ns. Conversely, a relative time delay of 1 ns in the two-dimensional image corresponds to approximately 0.4 mm of position distance. Each line has a length of approximately 120 mm (300 ns) and a resistance of ∼24 Ω.

The front and back sides of the MCP assembly, along with the delay-line anode wires and the holder, are biased through the feedthrough decouple (FT12) from [105], which connects the MCP front, back, anode wires, and holder with copper cables inside the chamber. The bias voltages applied to the front and back sides of the MCP, the holder electrode, and the delay-line anode wires are listed in Table 3. A bias voltage of 2400 V is applied between the front and back of the MCP, resulting in an overall gain of approximately $10^{7}$. The avalanched electrons leaving the MCPs induce a fast positive signal on the back/front side of the MCPs, which are collected by the signal wires of the X and Y delay lines, as shown in Figure 7. The reference wires, wound next to the signal wires, are biased with slightly different voltages (+36V) by a BA3 module [105] and are used to suppress electromagnetic noise picked up in the vacuum chamber. Five signals are read out: one from the MCP back or front side, two from the ends of the X delay line, and two from the ends of the Y delay line. The MCP back-side signal is inverted and then amplified twice by a photomultiplier amplifier (PM-AMP, Kaizu KN2104 [108]). The four anode signals are also amplified twice by the same PM-AMP.

During the calibration of the DLD system, the ion source typically used is either the vacuum gauge, which produces low-energy ions (around 100 eV), or the ^241^*Amα* sources placed above the mask on the chamber when supplying high voltage (HV) for the DLD system in ion mode, as shown in Table 3. In electron mode, since ions from the gauge rarely reach the MCP front surface with an applied voltage of 0 volts, the ^241^*Amα* sources are used instead.

The position at which the incoming particle impacts the MCP detector is determined by the time difference between the signals received from both ends of the same delay-line anode. The MCP position readout (X, Y) can be represented as follows:

(3) $$X=a_{x}\left(T_{X1}-T_{X2}\right)+b_{x},\;Y=a_{y}\left(T_{Y1}-T_{Y2}\right)+b_{y},$$

where $T_{X1},T_{X2},T_{Y1},$ and $T_{Y1}$ are the timings of the four delay-line anode signals in ns. The conversion factors from ns to mm, $a_{x}$ and $a_{y}$, can be calibrated using a collimated mask. Additionally, the timing offsets $b_{x},b_{y}$, which arise during pulse propagation and amplification, can be determined simultaneously. While this basic correction scheme, as described by Equation (3), cannot correct for rotations and non-linear distortions of the MCP image, it simplifies the implementation of the calibration algorithm. A typical setup for this calibration is illustrated in Figure 7.

Since the total length of the delay lines is constant, the sum of the propagation times from the charge impact position to both ends of the delay lines remains unchanged, regardless of where the event occurs. Therefore, the timing sums

(4) $$T_{Xsum}=T_{X1}+T_{X2},\;T_{Ysum}=T_{Y1}+T_{Y2}$$

are both expected to be constants, where $T_{X1}$, $T_{X2}$, $T_{Y1}$, and $T_{Y2}$ represent the timing signals from both ends of the two-dimensional delay lines relative to the MCP back-side signal, which serves as the trigger. When performing experiments involving position measurement or calibration of the DLD, a sum condition on the timing of the signals can be applied for event selection. This helps eliminate false triggers caused by electronic noise and pile-up events.

**Figure 8:**
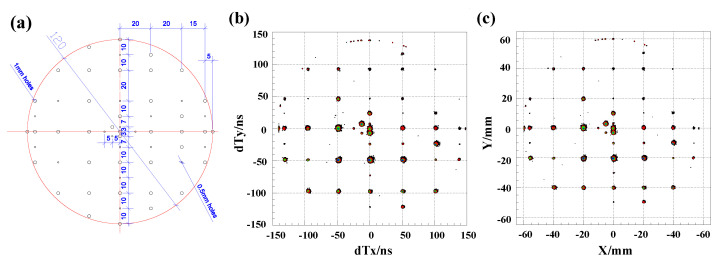
(**a**) CAD drawing of the effective area featuring ϕ0.5 mm or ϕ1 mm holes on the calibration mask. (**b**) Two-dimensional spectrum of raw signal imaging, displayed as a contour plot, based on the time difference in the x- and y-directions. (**c**) Calibrated two-dimensional position imaging spectrum of collimated ions passing through the mask, shown with contour display [36].

**Figure 9:**
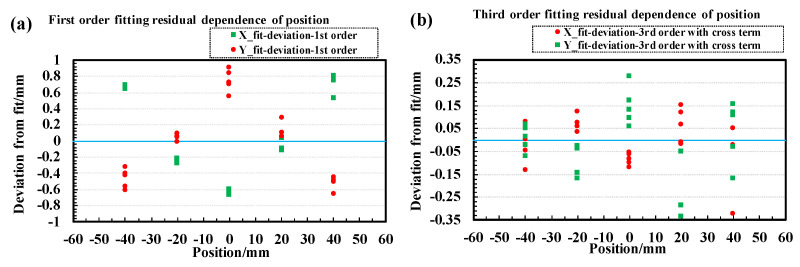
Fitting residual dependence of position for first-order calibration (**a**) and third-order calibration with cross terms (**b**) in fitting [36].

The physical positions ($X_{p}$, $Y_{p}$) of the hole points along the delay lines on the mask are determined from the designed values as illustrated in Figure 8a. A first-order calibration function similar to Equation (3) is constructed to correct the measured timing differences of each center positions ($dT_{X}$, $dT_{Y}$) on the holes to their physical positions. The parameters $a_{x},\;b_{x},\;a_{y},$ and $\;b_{y}$ from fitting are then applied to Equation (3) to calculate the calibration points using the measured timing differences from both ends of each delay line. To estimate the distortions in the MCP image, we calculate the first-order fitting deviations between the coordinates of the calibration points on the MCP image and their physical positions, as shown in Figure 9a. The mean absolute deviations are 0.44 mm and 0.42 mm for the X and Y directions, respectively, while the maximum deviations are 0.85 mm and 0.92 mm for the X and Y directions, respectively, without significant overall rotation of the mask when observing the entire MCP image. To achieve a much more precise and accurate calibration for the DLD system, we employ two-dimensional polynomials with 10 parameters for each hole. These polynomials transform the high-accuracy timing differences at each position ($dT_{X},dT_{Y}$) of the calibration points to their corresponding physical positions ($X_{p},Y_{p}$) as closely as possible. The correction functions are as follows:

(5) $$\begin{array}{c} X_{c}=p_{0}+p_{1}dT_{X}+p_{2}dT_{y}+p_{3}dT_{X}^{2}+p_{4}dT_{X}dT_{Y}\\ +p_{5}dT_{Y}^{2}+p_{6}dT_{X}^{3}+p_{7}dT_{Y}^{3}+p_{8}dT_{X}^{2}dT_{Y}+p_{9}dT_{X}dT_{Y}^{2} \end{array}$$

and

(6) $$\begin{array}{c} Y_{c}=q_{0}+q_{1}dT_{X}+q_{2}dT_{y}+q_{3}dT_{X}^{2}+q_{4}dT_{X}dT_{Y}\\ +q_{5}dT_{Y}^{2}+q_{6}dT_{X}^{3}+q_{7}dT_{Y}^{3}+q_{8}dT_{X}^{2}dT_{Y}+q_{9}dT_{X}dT_{Y}^{2}, \end{array}$$

where $dT_{X}=T_{X1}-T_{X2}$ and $dT_{Y}=T_{Y1}-T_{Y2}$ are the timing differences of the both delay-line signals for the X and Y layers. In Equations (5) and (6), ($X_{c},Y_{c}$) represents the corrected position of a calibration point with non-corrected timing difference ($dT_{X},dT_{Y}$), and $p_{i}$ and $q_{i}$ (*i* = 0–9) are parameters determined by fitting all the calibration points within an effective region (−45 mm ≤ *X* ≤ 45 mm, −45 mm ≤ *Y* ≤ 45 mm) of the mask and setting ($X_{c},Y_{c}$) to their physical positions. Regions near the outer edge of the MCP, which exhibit poor linearity, are excluded from the calibration analysis.

**Figure 10:**
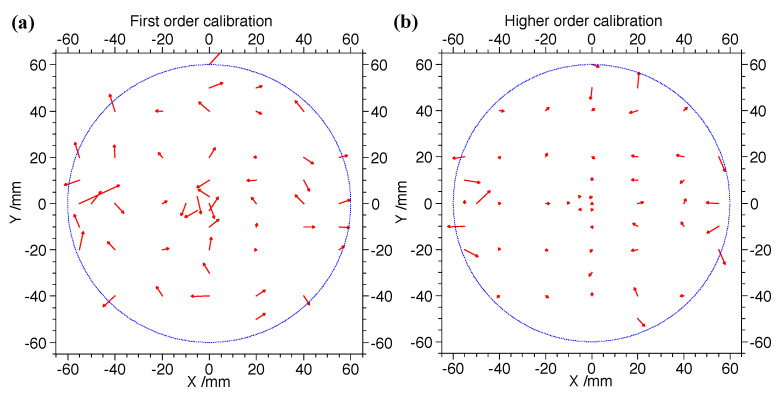
The vector field map of the correction vector $\vec{V_{ij}}$, derived from the difference between the expected and the calibrated/measured mean values for each hole center spot. (**a**) displays a first-order correction map, while (**b**) shows a higher-order correction map. For better visibility, the lengths (magnitudes) of the vectors are enlarged by a factor of 5. The edge of the active area of the DLD is indicated by the dashed blue circle [36].

To evaluate the performance of this position calibration method, we applied the calibration parameters from one calibration run to correct another dataset taken under the same conditions but was statistically independent from the calibration run. The deviations of x and y coordinates from their physical values were calculated. The distributions of the deviations along with their uncertainties of the measurements in the X- and Y- directions are shown in Figure 9b. The average absolute deviations (accuracy) for second-order or third-order corrections, with or without cross terms, amounted to ≤82 μm and ≤ 147μm for x and y coordinates, respectively, which are significantly smaller than those from the first-order correction, at approximately 0.4 mm. The root mean squares (RMS, resolution: $\sigma_{x}$, $\sigma_{y}$) of the higher-order deviation distributions were all ≤0.068 mm and 0.142 mm for both x and y coordinates. In this test, the maximum deviation was <0.4 mm, as shown in Figure 10. For the radial position resolution, we transformed the X and Y resolutions as follows:

(7) $$R_{xy}=\sqrt{{\sigma_{x}}^{2}+{\sigma_{y}}^{2}}.$$

Therefore, the radial position resolution was $R_{xy}$ = $\sqrt{0.68^{2}+0.142^{2}}$ = 0.157 mm. Comparing first-order and higher-order calibration methods revealed that higher-order calibration was necessary to accurately determine the position dependence of fitting deviations.

A correction vector $\vec{V_{ij}}$ was used for each spot to shift the measured values to the expected mean values.

(8) $$\vec{V_{ij}}=\left(X_{expected,ij}-X_{measured,ij}\right)\vec{e_{x}}+\left(Y_{expected,ij}-Y_{measured,ij}\right)\vec{e_{y}},$$

where $X_{expected,ij}$ and $Y_{expected,ij}$ are the expected X and Y values, while $X_{measured,ij}$ and $Y_{measured,ij}$ are the measured X and Y values, and $\vec{e_{x}}$ and $\vec{e_{y}}$ are the unit vectors in the X and Y directions. The resulting correction matrix $\vec{V_{ij}}$ adjusts each measured mean value to its expected position on the mask.

A 2D vector field figure, as shown in Figure 10, illustrates the correction vector $\vec{V_{ij}}$ for each spot on the mask, indicating shifts with their respective angles and magnitudes (line lengths). For better visibility, the lengths (magnitudes) of the vectors are enlarged by a factor of five. The edge of the active area of the MCP is marked by a dashed blue circle. As seen in Figure 10, the deviation amplitude of the third-order fitting is significantly smaller at each point compared to the first-order fitting, except for a few points near the edge of the delay lines. This confirms that higher-order correction is necessary for better accuracy. Additionally, no curl in the direction of the vectors is observed in the first-order fitting vector field map, suggesting that neglecting rotation in the X-Y plane is reasonable. From both the first- and higher-order correction maps in Figure 10, larger deviations are noticeable near the edge of the delay lines.

## 4. Experimental Test of the Detectors

To evaluate the performance of the detectors, including their timing and position resolving powers, efficiency, and active area, specific setups are required both offline with ion sources and online with RI beams. In this study, we used experimental tests on mirror-type foil-MCP detectors adopted from [30,36] to illustrate how to assess the performance of similar foil-MCP detectors. The conversion foil used in these tests was made of mylar (2 μm) coated with aluminum (∼0.1 μm). The distance from the accelerating grid to the foil was maintained at 8 mm, with grid wire spacing set at 1 mm.

### 4.1. Experimental Setup

#### 4.1.1. Offline Setup

**Figure 11:**
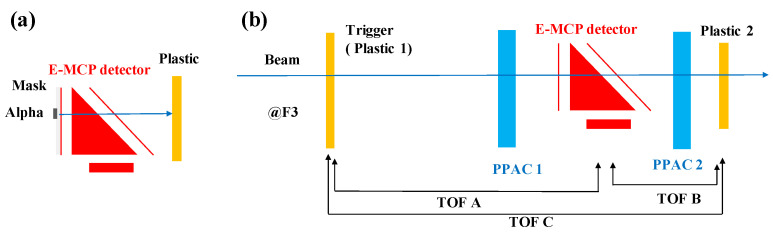
Schematic view of detector arrangement of the offline (**a**) experiment with α source and online (**b**) experiment with heavy-ion beams [36].

The offline test setup is illustrated in Figure 11a. A mask with multiple holes, each with a diameter of 1 mm or 0.5 mm, was designed to collimate the ^241^Am α source for testing the position resolution of the electrostatic mirror-type foil-MCP detector. Three ^241^Am α sources were affixed to the mask, which was secured to the foil-plate with screws. A plastic scintillation counter was positioned right after the E-MCP detector to capture the passing alpha particles. This counter comprised a plastic scintillator and two PMTs (model number H2431-50 [109]) attached to both ends of the scintillator. The coincidence signal from the left and right PMTs of the plastic scintillator served as the trigger for the CAMAC Data Acquisition (DAQ) system.

#### 4.1.2. Online Setup

An online experiment was conducted at the secondary beam line, SB2 course, at the Heavy Ion Medical Accelerator in Chiba (HIMAC) at the National Institute of Radiological Science (NIRS) [110,111], Japan. A primary beam of ^84^Kr^36+^ with an energy of 200 MeV/nucleon was used to evaluate the performance of the E-MCP detector. The right panel of Figure 11 provides a schematic view of the experimental setup. This setup includes two delay-line parallel plate avalanche chambers (PPACs) [41], one electrostatic MCP (E-MCP) detector, and two plastic scintillator counters. The position of each ion is determined and tracked by two gas-filled PPACs (PPAC1 and PPAC2), each measuring 100 mm × 100 mm, positioned between the two plastic scintillation counters. Each plastic scintillation counter comprises one scintillator and two PMTs (model number H2431-50 [109]) at both ends of the scintillator. The E-MCP detector is situated between PPAC1 and PPAC2. The position of each ion as it passes through the foil is reconstructed by the two PPACs.

### 4.2. Experimental Results of MCP Detectors

#### 4.2.1. Detection Efficiency

**Figure 12:**
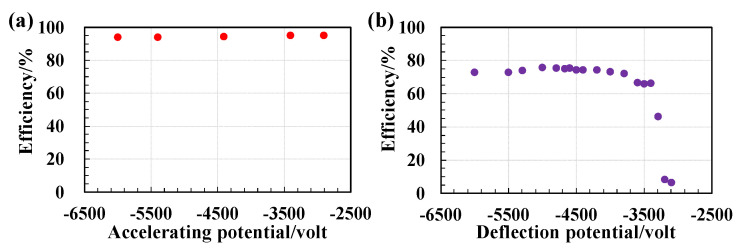
(**a**) The overall detection efficiency of the electrostatic MCP detector plotted against the deflection potential for ions of ^84^^Kr36+^. The ratio between the deflection and accelerating potentials is consistently maintained at approximately 0.79. (**b**) The efficiency of detecting α particles emitted from a ^241^Am source. The accelerating potential is held constant at −6000 V, while the deflection potential is adjusted throughout the process [36].

**Figure 13:**
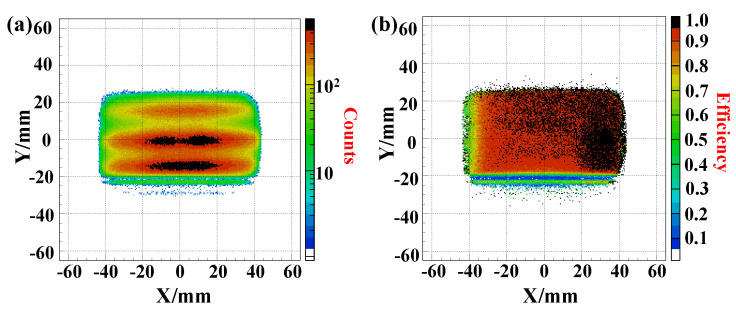
(**a**) shows the measured position distribution of beams by the electrostatic MCP detector. (**b**) indicates the local detection efficiency distribution of the detector [36].

Efficiency is a basic characteristic that indicates the probability of a specific type of particle successfully passing through a conversion foil and being detected by the DLD. Both offline and online tests measure this efficiency by comparing the total number of events recorded by the MCP detector to the number of gated trigger events. The MCP detector’s efficiency is validated using heavy-ion beams and alpha particles emitted from a ^241^*Am* source, with the findings presented in Figure 12 and Figure 13. Figure 12a illustrates a consistent efficiency of approximately 95% for heavy ions of ^84^Kr^36+^ as the deflection potential is varied, while maintaining a ratio of the deflection potential to the accelerating potential at about 0.79. In Figure 12b, the efficiency of detecting alpha particles from the ^241^Am source is shown as a function of the deflection potential, with the accelerating potential held constant at −6000 V and the deflection potential adjusted by altering the HV of the outer mirror grid. It is evident from Figure 12b that the detection efficiency declined significantly when the ratio of the deflection potential to the accelerating potential approached 0.5, which affected the mirror’s transparency to secondary electrons as described by Equation (A6). At the active area of the MCP, a stable efficiency of about 75% for detecting α particles from the ^241^Am source was achieved. The overall efficiency was determined by considering all events detected across the entire MCP detector relative to the events that occurred, under the assumption that the detection efficiency is uniform regardless of the detector’s detection location.

The MCP detector’s local detection efficiency was evaluated using three defocused beams that collectively span an area of approximately 90 mm by 50 mm. Figure 13a presents a two-dimensional (2D) histogram depicting the positions of the beams as recorded by the MCP detector. The local detection efficiency was represented in a 2D histogram format, with both the X and Y axes divided into bins of 0.5 mm each, as illustrated in Figure 13b. Efficiency was calculated by dividing the number of events detected by the E-MCP detector within each 0.5 mm bin by the number of gated trigger events. The results depicted in Figure 13b show minor variations in local efficiency. Notably, there is a pronounced deficiency in efficiency along the left edge. This efficiency drop may be attributed to an uneven gain factor across the MCP stack, and it can be mitigated by applying a higher voltage between the MCP layers to enhance the gain.

#### 4.2.2. Timing Resolution

Normally, for one time-of-flight (TOF) spectrum, the distribution contains the information of two timing detectors, which is characterized by the uncertainty σ or FWHM of the distribution. To determine the intrinsic timing resolution of the E-MCP detector, another timing detector has to be applied, as shown in Figure 11. Assuming that the timing distribution of each timing detector follows a Gaussian distribution, the timing resolution of an individual detector can be determined by using three sets of TOF between three timing detectors: TOF(A), TOF(B), and TOF(C). Typically, a single time-of-flight (TOF) spectrum contains data from two timing detectors, which is defined by the uncertainty of the distribution, denoted as σ or FWHM. To ascertain the intrinsic timing resolution of the MCP detector, it is necessary to incorporate an additional timing detector, as depicted in Figure 11. Given that the timing distribution for each detector is assumed to adhere to a Gaussian pattern, the timing resolution for a single detector can be determined by examining three distinct TOF measurements across three timing detectors, namely TOF(A), TOF(B), and TOF(C). The resolutions of three TOFs can be described as follows:

(9) $${\left(\sigma_{TOF(A)}\right)}^{2}={\left(\sigma_{pla1}\right)}^{2}+{\left(\sigma_{MCP}\right)}^{2},$$

(10) $${\left(\sigma_{TOF(B)}\right)}^{2}={\left(\sigma_{MCP}\right)}^{2}+{\left(\sigma_{pla2}\right)}^{2},$$

(11) $${\left(\sigma_{TOF(C)}\right)}^{2}={\left(\sigma_{pla2}\right)}^{2}+{\left(\sigma_{pla1}\right)}^{2},$$

where $\sigma_{pla1}$, $\sigma_{MCP}$, and $\sigma_{pla2}$ represent the intrinsic timing resolutions of the first plastic scintillator, the MCP detector, and the second plastic scintillator, respectively. The terms σ(TOF(A)) (or σ(A)), σ(TOF(B)) (or σ(B)), and σ(TOF(C)) (or σ(C)) refer to the “sigma” parameters obtained from fitting the three sets of TOF data. Utilizing these parameters, the intrinsic timing resolution of the E-MCP detector can be calculated by solving the following equation:

(12) $$\sigma_{MCP}=\sqrt{(\sigma^{2}\left(TOF\left(A\right)\right)+\sigma^{2}\left(TOF\left(B\right)\right)-\sigma^{2}\left(TOF\left(C\right)\right))/2},$$

where the $\sigma_{MCP}$ indicates the intrinsic timing resolution of the MCP detector.

The uncertainty $\delta (\sigma_{mcp})$ of the timing resolution $\sigma_{MCP}$ from the measurement can be expressed as follows:

(13) $$\delta \left(\sigma_{mcp}\right)=\sqrt{(\delta^{2}\left(\sigma \left(A\right)\right)\sigma^{2}\left(A\right)+\delta^{2}\left(\sigma \left(B\right)\right)\sigma^{2}\left(B\right)+\delta^{2}\left(\sigma \left(C\right)\right)\left(\sigma^{2}\left(C\right)\right)/\left(4\sigma_{MCP}^{2}\right)},$$

where δ(σ(A)), δ(σ(B)), and δ(σ(C)) are the uncertainties of the fitting “sigma” parameters, σ(TOF(A)) (or σ(A)), σ(TOF(B)) (or σ(B)), σ(TOF(C)) (or σ(C)), and the $\delta (\sigma_{mcp})$ indicate the uncertainty of the deduced value of the intrinsic timing resolution $\sigma_{MCP}$ of the E-MCP detector. The best obtained timing resolution is 43±2 ps in σ [35]. More details can be found in [35,36].

#### 4.2.3. Position Resolution

**Figure 14:**
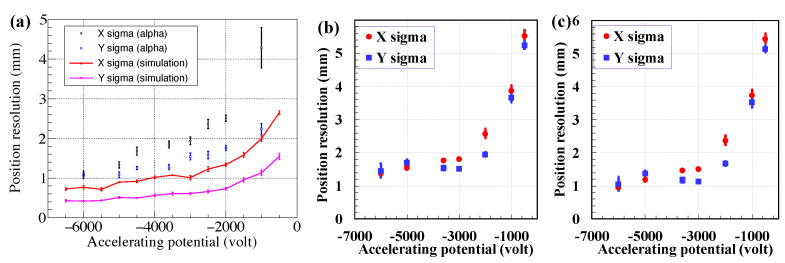
(**a**) Position resolution comparison of offline results (Mylar foil in “electron mode”) to simulation results as a function of the accelerating potential by keeping the ratio of accelerating potential and the deflection potential at ∼0.778. (**b**) Uncertainty of position measurement difference of the PPACs and the E-MCP detector as a function of accelerating potential. (**c**) Uncertainty of position measurement difference subtracted with the resolution of the PPAC system (assuming a resolution of 1 mm for two dimensions) as a function of accelerating potential [36].

The position resolution for the E-MCP detector in the offline test was checked by using collimated (hole sizes smaller than 0.5 mm in diameter) α from ^241^Am in front of the conversion foil. The black and blue data points in Figure 14a show the X- and Y-direction position resolutions as a function of accelerating potential, respectively. The ratio of accelerating potential and the deflection potential was kept at ∼0.778. The simulation result by SIMION [85] with the same setting of the HV supplies for the detector are indicated in solid line in Figure 14a. Red corresponds to the X-direction resolution and pink represents the Y-direction resolution. The differences of the experimental results and simulation results are mainly from the inhomogeneous electrostatic field, timing walk in electronics, spread of electrons in the MCP, and the initial condition difference of the real condition and simulation. It is obvious that by increasing the HV of the accelerating potential and the deflection potential, the resolutions of X and Y directions improve, which is consistent with the simulation results shown in Figure 14a.

**Figure 15:**
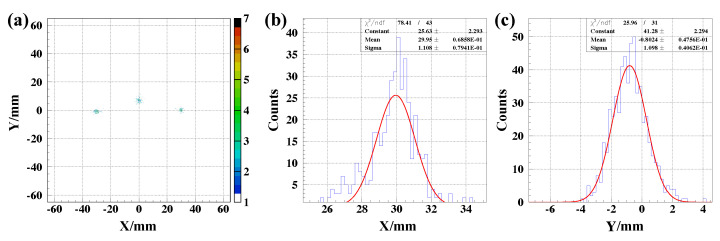
(**a**) shows the imaging of collimated α particles from three holes on a mask placed in front of the foil. (**b**,**c**) display the X- and Y-coordinate projections of the imaging from one hole. The Gaussian fitting parameter “sigma” of the peak is used to characterize the resolutions (X: 1.108 mm, Y: 1.098 mm). The deviations between the imaging points on the MCP detector and their corresponding physical positions on the mask are smaller than 1σ uncertainty (the resolution) of the measurements [36].

In one offline test run, we set three α sources (^241^Am) with nearly the same intensity of 4 MBq at the hole places of (−30 mm, 0 mm), (0 mm, 8 mm), and (30 mm, 0 mm) on the mask with hole sizes of less than 0.5 mm in diameter. The imaging of collimated α particles from the holes are shown in Figure 15a. The projections on the X and Y coordinates of the imaging of one hole are shown in Figure 15b,c, respectively. The uncertainty of the Gaussian fitting for position distribution on the X and Y coordinates were 1.108 ± 0.079 mm and 1.098 ± 0.041 mm, respectively. In this setting, the accelerating potential of −6000 V and deflection potential of −4668 V were supplied.

In the online experiment, the position on the foil with heavy ions passing though were measured by the E-MCP detector and also reconstructed by the PPACs. The uncertainty of position measurement difference of the PPACs and the E-MCP detector as a function of accelerating potential is shown in Figure 14b. The uncertainty of the position difference contains the intrinsic resolution of the E-MCP detector and the PPAC tracking system. To demonstrate the intrinsic resolution of the E-MCP detector, position resolutions of 1 mm for two dimensions of the PPAC tracking system were assumed to be subtracted from the uncertainty of the position difference. The derived intrinsic resolution of the E-MCP detector system, after subtraction of resolution of the PPAC tracking system, as a function of the accelerating potential, is shown in Figure 14c. The trend of position resolution as a function of the accelerating potential is consistent with those of the offline tests and simulations shown in Figure 14a.

## 5. Application of the Detectors at Nuclear Facilities

### 5.1. Utilization for Fragment Separator

Here, the usage of the foil-MCP detectors at the fragment separators BigRIPS at RIKEN [112] and the HFRS at HIAF [113] are discussed as examples [35,38]. The next-generation fragment separators, such as BigRIPS [35] and HFRS [38,114], consisting of two functional parts: the pre-fragment separator for selection and separation of RIs and the main-fragment separator for high-resolution PID of RIs. These fragment separators are primarily powerful separators, but can be also operated as high-resolution spectrometers simultaneously.

An achromatic system typically offers the best performance for spatial separation of mono-isotopic beams [38,114]. The ion-optical system of the HFRS is doubly achromatic at the final focal plane. Thin production targets could be employed and installed at PF0. A degrader at PF2 is shaped to preserve achromatism at the final focal plane MF4. HFRS will be employed as a two-stage separator: the first stage from PF0 to PF4 is used for separating of the nuclei of interest through a Bρ-ΔE-Bρ selection, and the second stage from PF4 to MF4 is used for PID of the RIs with a Bρ-ΔE-TOF method. The second stage can also be used to deduce velocity and momentum dispersions of RIs on an event-by-event basis. This similar scheme for BigRIPS is described in [35].

The trajectory of a fully stripped ion moving through a magnetic field *B* is influenced by its mass number *A*, its charge Q=Ze (where *Z* is the proton number and *e* is the electron charge), and its momentum *P*. This relationship can be expressed with the following equation:

(14) $$B\rho =\frac{P}{Q}=\frac{mv\gamma }{Q}=\frac{A}{Z}\frac{cm_{\mu }}{e}\gamma \beta ,$$

where m_μ_ ≈ 931.494 MeV is the atomic mass unit, c the speed of light, ρ the radius of curvature, β = v/c the relativistic factor, and γ = ${\left(1-\beta^{2}\right)}^{-1/2}$ the relativistic Lorentz factor. The secondary nuclei are produced by fragmentation at PF0, and their velocity remains nearly constant. Therefore, selecting in Bρ with the dipoles between PF0 and PF2 is then equivalent to selecting in A/Z.

The identification of the resulting secondary cocktail beam on an event-by-event basis is performed in the second part of the HFRS. The nuclei of interest are transmitted up to the doubly achromatic focal plane (MF4 at HFRS and S0 at BigRIPS, a beam focused in both the horizontal and vertical planes with no dependence of angle and momentum).

The PID of the secondary cocktail beams is accomplished by the Bρ-ΔE-TOF method in the fragment spectrometer, which ensures the tagging of every particle delivered in the form of a cocktail beam from projectile or in-flight fission fragments. At the secondary stage, from PF4 to MF4 [35] (F3 to S0 [35]), the PID provides the atomic number *Z* and mass-to-charge ratio A/Q of a fragment by measuring energy loss, magnetic rigidity, and time of flight (ΔE-Bρ-TOF) with the corresponding beam-line detectors. The Bρ and TOF of RIs could be measured with the MCP detectors designed in this paper, which offer relatively high position and timing resolutions. The energy loss of RIs can be measured by an ionization chamber (IC).

The TOF between two foci (PF4 to MF4 at HFRS [35], F3 to S0 at BigRIPS [35]) could be measured by the corresponding MCP detectors: TOF = L/(β c), which can be used for velocity (β) measurements with a correction by the precision Bρ measurement with a MCP detector at a dispersive focus of MF2 at HFRS and F6 at BigRIPS. From Bρ= $\frac{P}{Q}$ = $\frac{mv\gamma }{Q}$= $\frac{A}{Q}\frac{cm_{\mu }}{e}\gamma \beta$, we can obtain the following: $\frac{A}{Q}=\frac{B\rho }{\beta \gamma }\frac{e}{m_{\mu }c}$. The atomic number Z of a heavy ion is deduced based on the energy loss of the charged particle in the IC.

The variable ΔE represents the energy losses in the IC, while the velocity β of the heavy ion in the IC can be derived from the TOF measured with MCP detectors between the PF4 and MF4 [38] (F3 and S0 [35]) focal planes. The position-sensitive detector in the focal plane MF2 of HFRS (F6 of BigRIPS), with its high momentum resolution, can be used to determine the Bρ/β spread of each fragment before it is injected into the storage ring SRing [38] (Rare-RI Ring [35]). Precise Bρ determination is achieved through trajectory reconstruction, where measured particle trajectories are combined with ion-optical transfer matrix elements deduced from experimental data from the MCP detectors [38] (or combined with scintillators [35]). A correlation of measured beam trajectories at the initial and final focal planes provides us with direct information of the ion-optical transfer map elements. For example, a correlation between the final position (MF4 *x*) and the initial angle (PF4 *a*) provides us with a measurement of the (x|a) element of the transport map. In addition to high-resolution PID of RIs, with the beam-line trajectory monitoring and deduction of the ion-optical transfer matrix elements, a fast-response tuning method could be established to help overcome the difficulties caused by low intensity, and large longitudinal and transverse emittances of the exotic RI beams.

### 5.2. Utilization for a New Scheme of Mass Measurements

For HIAF, the development of high-performance position-sensitive and timing detectors enables the establishment of the IMS method at the storage ring SRing and the Bρ-TOF method on the beam line simultaneously. Low energy loss and minimal energy straggling of RIs in these detectors are crucial for accurate and precise momentum/velocity reconstruction, which is essential for in-ring mass deduction and beam-line Bρ-TOF mass measurements. With the advancement of the foil-MCP detector for simultaneous precise position and timing measurements, as described in [38], a new mass measurement scheme at HIAF could be realized. This scheme would allow for the execution of two complementary methods—Bρ-TOF mass measurements with the beam-line HFRS and IMS mass measurements via the storage ring SRing— in a single experimental run. At RIKEN, the implementation of the two complementary methods—Bρ-TOF mass measurements with the beam-line BigRIPS-OEDO and IMS mass measurements via the Rare-RI Ring—in a single experimental run was detailed in [35].

#### 5.2.1. Mass Measurements by Bρ-TOF Method with Beam Line

**Figure 16:**
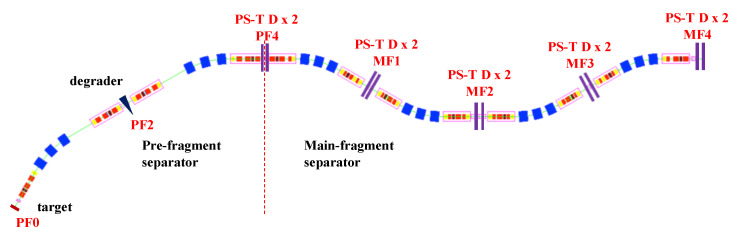
The potential completion status of the foil-MCP detector installation at HFRS. Dual foil-MCP detectors, indicated by purple blocks, could be used for measuring the position, angle, and timing of arrival of RIs at the foci (PF4, MF1-4) of HFRS on an event-by-event basis [38]. The ‘PS-T D’ refers to the position-sensitive timing detector.

The Bρ-TOF technique for mass measurements of exotic nuclei relies on measuring the Bρ and the corresponding TOF along the beam line with a length of *L* of the ion: $B\rho =\frac{\gamma m}{q}\frac{L}{TOF}$. The mass-to-charge ratio (m/q) can be expressed as follows:

(15) $$m/q=\frac{B\rho }{\gamma L/TOF}=B\rho \sqrt{{\left(\frac{TOF}{L}\right)}^{2}-{\left(\frac{1}{c}\right)}^{2}}$$

where *c* is the speed of light and γ is the Lorentz factor. The TOF can be determined with high precision using the MCP detectors at PF4 and MF4, as illustrated in Figure 16. However, the precision of the resultant mass is significantly limited by the measurements of Bρ at MF2 and the flight length *L* from PF4 to MF4. Therefore, in practice, nuclei with well-known masses are measured alongside nuclei with unknown masses to calibrate the relationship between TOF, Bρ, and mass.

The derived mass uncertainty from Equation (15) is described as follows:

(16) $$\frac{\delta m}{m}=\frac{\delta (B\rho )}{B\rho }+\frac{1}{1-{\left(\frac{1}{c}\frac{L}{TOF}\right)}^{2}}\left(\frac{\delta (TOF)}{TOF}-\frac{\delta L}{L}\right)$$

The mass resolving power of this technique is highly influenced by the resolutions of the MCP detector (specifically, the TOF from PF4 to MF4 and the position measurement at MF2) and the momentum resolution of the beam line, which is determined by the ion-optical design. Bρ-TOF mass measurement with a typical TOF ∼1 μs yields mass data with an accuracy ranging from $10^{-4}$ up to the level of $10^{-6}$ depending on the statistical data. This technique enables the simultaneous measurements of numerous nuclides, including reference isotopes and isotopes of interest.

#### 5.2.2. Mass Measurements with Storage Ring Mass Spectrometry

The storage rings ESR, CSRe, and Rare-RI Ring, designed for mass measurements using isochronous mass spectrometry (IMS) at the nuclear facilities GSI [21,22,23], IMP [17,26], and RIBF [32,104,112], have been utilized with projectile and fission fragments. This application has led to numerous significant findings in nuclear physics and astrophysics, based on the precise mass determinations achieved through IMS. Storage ring spectrometers are highly effective devices for achieving high-accuracy mass measurements (10^−6^–10^−7^) of very short-lived isotopes. Recent advancements, such as the in-ring double-TOF method at CSRe/IMP and CRing/FAIR, in the measurements of velocity techniques have extended the applicability and ability of storage ring mass spectrometry [38,103].

The SRing is an essential part of the HIAF and it is a dedicated storage ring for precision mass and half-life measurements of short-lived nuclei with life-times down to several tens of microseconds [38,103]. The basic principle for storage ring mass spectrometry describing the relationship between the mass-over-charge ratio (m/q) and the revolution period (T) or the revolution frequency (f) can be quantitatively expressed in first-order approximation:

(17) $$\frac{dT}{T}=-\frac{df}{f}=\frac{1}{{\gamma_{t}}^{2}}\frac{d(m/q)}{m/q}-\left(1-\frac{\gamma^{2}}{\gamma_{t}^{2}}\right)\frac{dv}{v}=\frac{1}{\gamma^{2}}\frac{d(m/q)}{m/q}+\left(\frac{1}{\gamma_{t}^{2}}-\frac{1}{\gamma^{2}}\right)\frac{d(B\rho )}{B\rho },$$

where γ is the relativistic Lorentz factor, v the velocity of the ion, and Bρ the magnetic rigidity. $\gamma_{t}$ is the so-called transition energy of the ring, where the momentum compaction factor $\alpha_{p}=\left(\Delta C/C\right)/\left(\Delta B\rho /B\rho \right)$ represents the ratio between the relative change in orbital length of an ion stored in the ring and the relative change in its magnetic rigidity. The transition energy of storage rings, $\gamma_{t}$, is defined by $\alpha_{p}\equiv 1/\gamma_{t}^{2}$. Based on this principle, two complementary experimental methods, namely Schottky (SMS) and IMS, have been developed for accurate mass measurements [17]. One method is to set the Lorentz factor γ of a specific ion species to the ring’s transition energy $\gamma_{t}$, so that $\gamma =\gamma_{t}$. This achieves the isochronous condition. Under this condition, the revolution period *T* of the ions depends solely on their mass-to-charge ratio m/q, regardless of their momentum spreads. This property is only valid within a limited mass-to-charge region known as the isochronous window [16]. For ion species outside the isochronous window, or even within it, the isochronous condition is not perfectly met. This results in an unavoidable momentum spread due to the acceptance of the storage ring, which broadens the distribution of the revolution period and reduces mass resolving power. To minimize the spread of the revolution period, it is essential to correct the Bρ spread of stored ions. However, directly measuring the Bρ of each ion, especially those with unknown m/q, is challenging due to the definition of Bρ.

To reduce the spread of the revolution period, precise Bρ or β measurements of stored ions are necessary. It is assumed that ions with the same magnetic rigidity will follow the same closed orbit, regardless of their species. Therefore, correction of the magnetic rigidity can be achieved by adjusting the corresponding orbit. In IMS experiments at ESR, this is verified with the momentum acceptance limited by a pair of slits (Bρ-tagging) at a dispersive plane in FRS, which results in a significantly improved resolving power [16]. However, the transmission efficiency of the rare exotic nuclides is reduced dramatically by this approach, limiting its application for nuclides far from the valley of stability, which have very tiny production rates. This limitation can be overcome by the measurement of the velocity or momentum of each stored ion before injection into the ring or inside the ring. One method (double-TOF) to measure the revolution time with extra determination of the velocity is by using two TOF detectors installed at the straight section of the storage rings. The original idea was first proposed at GSI [115] and realized at CSRe in IMP [116]. The Collector Ring (CR) at the next-generation facility FAIR [117] and the Spectrometer Ring (SRing) at HIAF [113] will both be equipped with double-TOF detectors [25] at the straight sections. Another method (in-ring Bρ-TOF) is the measurement of the ion’s position in the dispersive arc section of a storage ring by using a position-sensitive low-energy-loss foil-MCP detector or a non-destructive position-sensitive cavity doublet to determine the ion position event by event to realize the correction by magnetic rigidity measurement. The SRing is proposed to measure the exotic nuclei in IMS mode with additional velocity or magnetic rigidity measurement for TOF correction of non-isochronous RIs. Therefore, the correction of the in-ring revolution time by Bρ (equivalent to β for a certain close orbit) measurements with double-TOF detectors can be carried out. Alternatively, to realize the correction of the non-isochronous TOF of RIs in the SRing, a foil-MCP detector with high position and timing performance at the same time, which is described within this paper, could be installed at a dispersive arc section of the SRing to measure the ion’s position to deduce the Bρ of each ion event by event. A foil-MCP detector can be installed at a dispersive arc of the SRing where the value of horizontal dispersion function D_*x*_ is large in order to deduce the Bρ value precisely [38].

Compared to the double-TOF method, in principle, only one foil-MCP detector with relatively lower energy loss of the circulating RIs can realize the same correction effect. Actually, the double-TOF detectors can be replaced by one compact detector with high performance in both good timing and position measurements to reduce the accumulated energy losses of RIs when passing through the foil of the detector for hundreds to thousands of turns.

In summary, the Bρ-TOF method would be a perfect marriage with multi-physics experiments in the next-generation RI beam facility HIAF in China. To reach the most exotic area of the chart of nuclides and to have an effective use of the precious beam time, two complementary TOF techniques for mass measurements can be employed at HIAF in one experimental run. One is the well-developed Bρ-TOF [5] method with the beam-line HFRS and another is the IMS method via the SRing with extra Bρ (or β) measurements event by event.

For the Rare-RI Ring, the mass-to-charge ratio m_1_/q_1_ for the nucleus with unknown mass could be deduced from the known mass m_0_/q_0_ with extra Bρ/β measurements expressed as follows:

(18) $$\frac{m_{1}}{q_{1}}=\frac{m_{0}}{q_{0}}\frac{T_{1}}{T_{0}}\sqrt{\frac{1-\beta_{1}^{2}}{{1-{\left(\frac{T_{1}}{T_{0}}\right)}^{2}\beta_{1}}^{2}}}=\frac{m_{0}}{q_{0}}\frac{T_{1}}{T_{0}}\sqrt{\frac{1-{\left(\frac{T_{0}}{T_{1}}\right)}^{2}}{{\left(\frac{m_{0}}{q_{0}}c{{\left(B\rho \right)}_{0}}^{-1}\right)}^{2}}+1},$$

where m is the mass of the ion, q is the charge of the ion, Bρ is the magnetic rigidity, and B is the magnetic field of the ring. T_0_ and T_1_ are the revolution times of an ion with a known mass, and the relativistic factor β = v/c denotes the particle velocity relative to the velocity of light c. When a particle of interest with a mass-to-charge ratio m_1_/q_1_ has the same momentum as that of a reference particle with a mass-to-charge ratio m_0_/q_0_, the flight path length of these particles become identical in the isochronous storage ring. m_1_/q_1_ can be deduced from the ratio m_0_/q_0_ of the reference nucleus with the revolution time (T) and velocity (β)/momentum (Bρ) measurements of all ions. The parameter K = $\sqrt{\left(1-\beta_{1}^{2}\right)/\left({1-{\left(\frac{T_{1}}{T_{0}}\right)}^{2}\beta_{1}}^{2}\right)}$ and P = $\sqrt{\left(1-{\left(\frac{T_{0}}{T_{1}}\right)}^{2}\right)/{\left(\frac{m_{0}}{q_{0}}c{{\left(B\rho \right)}_{0}}^{-1}\right)}^{2}+1}$ are defined as the velocity correction factor and momentum correction factor, respectively.

Based on the technique of IMS, the isochronous condition can only be fulfilled exactly for one species of ion. The revolution times of the other kind of ions still depend on their velocity or momentum. The non-isochronicity effect can be corrected with the velocity (uncertainty ∼$10^{-4}$) or momentum (uncertainty ∼$10^{-4}$) of each ion determined in addition to the revolution time (uncertainty ∼$10^{-6}$). At the Rare-RI Ring, a new method is utilized: the total TOF of an ion inside the ring is measured by the TOF detectors at its injection as a start TOF (using a foil-MCP detector with low energy loss) and at the extraction part as the stop TOF outside the ring. The velocity and momentum of the injected ions can be measured before its injection by the foil-MCP detector at a dispersive plane with low energy loss and small angular scattering of the ions to ensure the accuracy of velocity and momentum measurements. The BigRIPS-OEDO-SHARAQ and the injection beam line of the Rare-RI Ring is originally designed for the particle selection, high efficient transmission, and particle identification of secondary ions from in-flight fission or projectile fragmentation reactions. The possibility of employing the BigRIPS-OEDO or BigRIPS-OEDO-SHARAQ beam line for mass measurements via the Bρ-TOF technique is highly motivated by its high resolution at a dispersive focus (76 mm/% or 147 mm/%). TOF measurements at two achromatic foci of the high-resolution beam line, along with additional Bρ corrections, enable the Bρ-TOF method. This allows for mass measurements within the same experimental run, combined with extended in-ring IMS mass measurements, utilizing Bρ/momentum and timing information at one focal plane in two different approaches [35,38,103]. Foil-MCP detectors can be versatile instruments which can be used on the beam line for two-dimensional position measurements to reconstruct beam trajectory, and for beam-line momentum measurements for velocity reconstruction. Meanwhile, it can be used for position monitoring and revolution time measurement turn by turn inside the storage ring, R3. Low energy loss and small angular straggling of the detected incident ions are indispensable for reconstruction of the velocity for in-ring mass deduction or momentum measurement with good accuracy and high precision for Bρ-TOF mass measurements. High-resolution TOF measurement in ring is not only significant for identification of nuclei with high resolving power but can also be used for mass measurements directly. The fast low-energy-loss position-sensitive timing detector described within this paper will be utilized both in-ring and on the beam line to achieve higher performance mass measurements, simultaneously employing the Rare-RI Ring in conjunction with the high-resolution beam lines BigRIPS-OEDO-SHARAQ by the two complementary TOF Methods: IMS and Bρ-TOF.

### 5.3. The Position-Sensitive Detector Measures the Betatron Function and Dispersion Function in the Ring

An example of the application of position-sensitive foil-MCP detectors within heavy-ion storage rings is the measurement of the betatron function and dispersion function within the ring [103]. The relative momentum deviation of the ion relative to the reference ion in a storage ring is as follows: $\delta =\Delta P/P_{0}=\left(P-P_{0}\right)/P_{0}$. The momentum of a reference ion with an initial momentum of $P_{0}$ relative to $x_{0}$ can be expressed as follows: $x\left(s\right)=x_{\beta }\left(s\right)+x_{\delta }\left(s\right)$. The transverse motion of a particle is the sum of betatron motion (homogeneous part), $x_{\beta }=\sqrt{\left(\varepsilon_{x}\beta_{x}\right)}cos\left(\varphi_{x}+\varphi_{0}\right)$, and a displacement because of the momentum dispersion (inhomogeneous part), $x_{\delta }=D\left(s\right)\delta$. As betatron motion is the homogeneous part, the averaged position of the beam can be described as follows:

(19) $$x=x_{0}+x_{\beta }+x_{\delta }\left(s\right)=x_{0}+\sqrt{\left(\varepsilon_{x}\beta_{x}\right)}cos\left(\varphi_{x}+\varphi_{0}\right)+D\left(s\right)\delta ,$$

where $x_{0}$ is the center of the reference ion (typically 0 mm).

Figure 5 displays the position-angle information (*x*,$x^{\prime }$) of each turn of the ^38^*K*^19+^ ion as measured by the simulated position-sensitive probe in the ring with COSY and MOCADI. Figure 5c shows data from a position-sensitive detector without energy loss (such as a Schottky probe), while Figure 5d shows data from a position-sensitive detector with energy loss (foil-MCP detector). Using the periodic dispersion data, the necessary conditions for high-resolution mass measurements can be achieved. The curve shapes are influenced by the betatron functions, allowing the Brho-value to be determined with an accuracy better than 10^−4^. The function used to fit the betatron oscillation is as follows: $x=x_{0}+a\cdot exp\left(-b\cdot T\right)\cdot sin\left(2\pi \cdot \left(T-T_{0}\right)/\omega \right)+c\cdot T$, where *x* represents the position on the foil, *T* is the measured turn number, and $T_{0}$ is the phase shift of the betatron oscillation. The fitting constant $x_{0}$ accounts for the position on the detector foil due to the deviation in Bρ of the particle. The parameter *a* denotes the amplitude of the betatron function, *b* describes damping and scattering effects, *c* is determined by the energy loss in the detector foil, and ω is the angular frequency of the betatron function. The reconstructed dispersion function ($D_{x}$) can be accurately produced with a precision of approximately $10^{-4}$ from a simulation study [103] of the foil-MCP detector position in the storage ring, as illustrated in Figure 5b,d. Detailed simulations are summarized in [103] and will be made available for open access in [118], including detailed applications, for the scientific community in the future.

## 6. Discussion and Outlook

In this review, we outlined various types of foil-MCP detectors used in nuclear physics facilities, including their operating principles, design, specifications, characteristics, test method introduction using experimental results, and performance through simulations. MCP detectors equipped with a thin foil for measuring SEs are efficient tools for high-precision position and timing measurements. These detectors are versatile instruments that can be used on the beam line for two-dimensional position measurements to reconstruct beam trajectories for tuning, high-resolution particle identification (PID), beam-line momentum measurements of heavy ions for velocity reconstruction, and beam-line TOF measurements between two foci to ensure high-resolution PID and deduce the velocity of each radioactive ion. Additionally, they can be used for position monitoring and revolution time measurements turn by turn inside the storage rings for direct mass measurements, in-ring Betatron function reconstruction, and dispersion function measurements. A new scheme of mass measurement techniques, combining IMS and Bρ-TOF methods in a single experimental setting using these detectors, has been summarized. Furthermore, the potential applications of these detectors in Penning trap and MR-TOF mass spectrometries for efficiency enhancement, alignment, and resolving power improvement have been briefly discussed. These promising new mass measurement methods as discussed in the article could enable the measurement of the most exotic nuclei, approaching the drip lines far from the valley of stability, which have been challenging to study due to their low production rates. The high-accuracy and high-precision mass values will be significant inputs for modeling explosive nucleosynthesis processes (such as r-, rp-, and νp-processes), helping to illuminate the origin of elements and the evolution of stars in our universe. The enhanced efficiency and advanced capabilities of these detectors will pave the way for new discoveries in nuclear physics, broadening the chart of nuclei.

## Figures and Tables

**Table 1:** Timing resolution and X, Y resolution dependence on distance between C-foil and MCP for the B×E-MCP detector, by SIMION simulation.

Distance Between C-Foil and MCP	1 mm	2 mm	3 mm	4 mm
Timing resolving power (σ, ps)	16	20	16	16
X-resolution (FWHM, mm)	0.15	0.40	0.38	0.69
Y-resolution (FWHM, mm)	7.68	6.86	7.00	6.43

**Table 2:** The corresponding changes in the simulated two-dimensional position (X and Y) and timing resolutions of the MCP detector when the electric field and magnetic field strength change. The electric and magnetic fields are arranged in parallel for the detector.

Electric Field (Volt)	1000	1000	2000	1000	2000	1000	1000	1000
**Relative Initial Magnetic Field (Gauss)**	**0**	**212**	**212**	**864**	**864**	**529**	**2115**	**9520**
Timing resolution (σ, ps)	400	490	233	476	237	410	441	466
X-resolution (FWHM, mm)	9	0.23	0.29	0.06	0.08	0.09	0.037	0.006
Y-resolution (FWHM, mm)	9	0.25	0.30	0.06	0.09	0.09	0.040	0.007

**Table 3:** Typical high-voltage settings for delay-line MCP detector.

	Ion Detection Mode	Electron Detection Mode
MCP front	−2400 V	0 V
MCP back	0 V	+2400 V
Anode holder	0 V to +250 V	+2400 V to +2650 V
Reference wires	+250 V	+2650 V
Signal wires	+286 V	+2686 V

**Figure A1:**
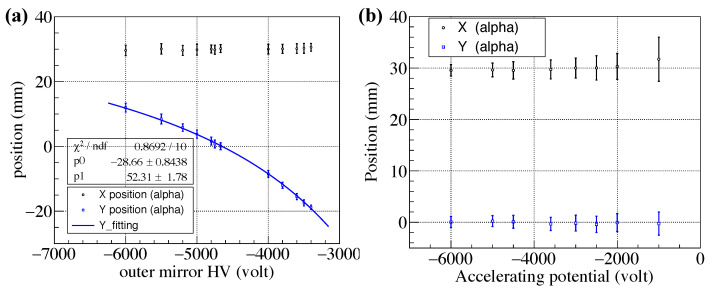
(**a**) shows the X, Y positions of the SEs deflected onto the MCP by the electrostatic detector deduced from the collimated α source at position (30 mm, 0 mm) on a mask with a hole size of ∼0.5 mm in diameter, as a function of outer mirror potential while keeping the accelerating grid HV of −6000 V. (**b**) displays the X, Y positions of the imaging of the same collimated hole as a function of accelerating potential by keeping the ratio of potential of the deflection potential to accelerating potential as a constant [36].

## Data Availability

No original data available in this review.

## Appendix A. Electromagnetic Motion of Secondary Electrons

### Appendix A.1. Electrostatic Mirror Detector

The electromagnetic force created by an electric field $\vec{E}$ and a magnetic field $\vec{B}$ on a charged particle with charge q to bend and direct the SEs can be given as follows:

(A1) $$\vec{F}=m\vec{\ddot{r}}=\vec{E}q+q\vec{v}\times \vec{B}$$

To achieve high timing and spatial resolution for the electrostatic mirror detector, careful calculations of the relationships between the motion of SEs inside the detector and potentials supplied for the plates of the detector are required. In order to perform the calculations, the whole system, consisting of the conversion foil, accelerating grid, the mirror grids, and the MCP detector is subdivided into three regions (see Figure 2a). The first one extends from the accelerating grid to the inner mirror front side, which is a field-free part. The second one is between the inner and outer mirror surfaces, where a homogeneous electric field is assumed. The third region is from the front side of the inner mirror to the MCP front, which is assumed to be field-free too. Three particles, starting from the different points and in the same direction but with different energies, are traced inside the system. As magnitudes of potentials of several kV are applied to potential plates or grids to accelerate or bend the SEs, the whole system can be described non-relativistically. The typical initial energy of SEs is several eV (most of them), and in this section, we neglected the initial energy of SEs for calculation. After acceleration from the foil to the accelerating grid as depicted in the upper panel of Figure 2a, SEs gain a velocity of $v_{0}$. At a certain time, when the velocity component parallel to the mirror of the SE is decelerated to zero, and the electron reaches the maximal depth y inside the mirrors, measured from the entrance mirror grid plane, the following holds:

(A2) $$y=\cos\alpha^{2}D\frac{U_{acc}}{U_{mir}}.,$$

where $U_{acc}$ and $U_{mir}$ are, respectively, the acceleration potentials between the conversion foil and the accelerating grid, and the mirror deflection potential between the mirrors; D is the distance of the mirrors; and α is the tilted angle of the mirrors. When escaping the mirror, the electron gains velocity and continues freely drifting towards the MCP detector through the field-free region. As demonstrated in Figure 1a, *x* and *y* are the parallel and vertical path lengths of SEs in the mirror parts, and t is the shift of SEs along the Z-direction inside the mirror, satisfying the equations:

(A3) $$x=2D\sin\left(2\alpha \right)\frac{U_{acc}}{U_{mir}},$$

(A4) $$t=x\sin\alpha =2D\sin\alpha \sin\left(2\alpha \right)\frac{U_{acc}}{U_{mir}}.$$

For the convenience of calculation and design, the α is set to be $45^{\circ }$. When substituted for α with $45^{\circ }$, the following equations for *x*, *y*, and *t* are obtained:

(A5) $$x=2D\frac{U_{acc}}{U_{mir}},\;y=\frac{D}{2}\frac{U_{acc}}{U_{mir}},\;t=\sqrt{2}D\frac{U_{acc}}{U_{mir}}.$$

In principle, based on Equation (A5) and the condition D≥y, it is obvious that the electrostatic mirror will reflect the SEs to the MCP front only if the following condition is satisfied:

(A6) $$\frac{U_{mir}}{U_{acc}}\geq 0.5.$$

From the above analytical calculation of the motion of SEs inside the detector, the imaging position of the SEs induced by heavy ions from the foil onto the MCP front surface and total TOF inside the detector can be determined by the acceleration potentials $U_{acc}$ and the mirror deflection potential $U_{mir}$ added to the detector potential plates. Therefore, the position and timing information of the heavy ions when bombarding the foil can be reproduced by detecting the position and timing of SEs at the MCP detector. A simulation of the trajectory of SEs in the detector is shown in Figure 1a.

#### Appendix A.1.1. Isochronous Condition

To achieve high timing resolution, an isochronous condition [34,35,51] is considered. The total time of flight of three parts: the foil to the inner mirror wires, bending path between the inner and outer mirror wires, and free drift region from the inner mirror wires to the MCP surface. The total TOF of the probed particles (initial velocity of SEs considered to be $v_{0}\delta$) after exact solution of the equations of motion is given as follows:

(A7) $$\begin{array}{c} T=T_{0}+\left(\mp \frac{L_{1}+L_{2}-\sqrt{2}D\frac{U_{mir}}{U_{acc}}}{v_{0}}\pm \frac{2m_{e}v_{0}D\sin\alpha }{e\Delta V}\right)\delta , \end{array}$$

where $L_{1}$ and $L_{2}$, as shown in Figure 1, correspond to the length from the conversion foil to the incident point of SEs at the inner mirror plate along the Z-direction, and the length between the incident point of SEs at the inner mirror plate with the MCP front surface along the Y-direction. To make the second term of the right part of Equation (A7) zero, we obtain the isochronous condition in which the total TOF has no dependence on the initial velocity variation of SEs: $D/\left(L_{1}+L_{2}\right)=0.236\frac{U_{mir}}{U_{acc}}$. This relation enables us to optimize the foil acceleration and mirror deflection potentials such that the SEs reach the MCP isochronously.

#### Appendix A.1.2. Trajectory Confirmation of SEs by Experimental Data

The equation are demonstrated in Equation (A5) indicating that the position of the Y-direction of the imaging as functions of the accelerating potential and deflection potential. The X, Y direction position of the imaging by varying outer mirror HV and keeping the accelerating potential at −6000 V are drawn at Figure A1a and the Y-direction motion of the imaging is fit with $y_{exp}=P0/2\cdot \frac{U_{acc}}{U_{mir}}+P1$ (from Equation (A5)), where the parameters P0 represents the distance between mirror grids of 28 mm in design and P1 indicates the shift of the SEs in the mirror along beam direction. Trajectory and mirror distance can be well reproduced as shown in Figure A1a with the P0 value of 28.66 ± 0.84 mm and P1 value of 52.31 ± 1.78 mm, which are very close to the designed value of 28 mm and 52.672 mm (47.672 mm designed value and 5 mm shift in analysis code), respectively. Figure A1b shows the imaging position of X and Y coordinates of the collimated hole (30 mm, 0 mm) on DLD. As the ratio of the deflection potential to the accelerating potential is kept as a constant of 0.778 and the X, Y directions of the imaging will be the same for all the settings by varying the deflection and accelerating potential at the same time.

### Appendix A.2. Electrostatic and Magnetic Fields Crossly Arranged Detector

Write Equation (A1) in the Cartesian linear coordinate system as a matrix as follows:

(A8) $$\vec{F}=q\left\{\left(\begin{array}{c} E_{x}\\ E_{y}\\ E_{z} \end{array}\right)+\left(\begin{array}{c} v_{x}B_{z}-v_{z}B_{x}\\ v_{z}B_{x}-v_{x}B_{z}\\ v_{x}B_{y}-v_{y}B_{x} \end{array}\right)\right\}$$

The trajectories of the SEs in this coordinate system under the action of the electromagnetic field force are shown in Figure 1. Ideally, the SEs generated by heavy-ion bombardment on the surface of the conversion film (generally carbon film) are isochronously transported from the conversion film to the MCP surface under the action of an orthogonal uniform electromagnetic field.

As shown in the schematic diagram of the principle (see Figure 2c), the z-axis direction is perpendicular to the surface of the conversion foil, and the ions move through the conversion foil along the z-axis. The direction of the electric field and the direction of the magnetic field are along the negative direction of the z-axis and the positive direction of the y-axis, respectively. The distance between the center of the conversion foil and the center of the MCP detector is denoted by *D*. The magnitude of the electric and magnetic fields can be expressed in vector coordinates as follows:

$E=(0,0,-E_{z})$, $B=(0,B_{y},0)$. Then the formula (A8) can be reduced to the following:

(A9) $$F_{x}=qE_{x}-v_{z}B_{y},F_{y}=qE_{y},F_{z}=q\left(-E_{z}+v_{x}B_{y}\right)$$

The formula (A1) can be written as follows:

(A10) $$m\frac{d^{2}x}{d^{2}t}=-q\frac{dz}{dt}B,m\frac{d^{2}y}{d^{2}t}=0,m\frac{d^{2}z}{d^{2}t}=q\frac{dx}{dt}B-qE$$

Let us assume that the initial position of an electron is (x(0), y(0), z(0)) and its initial velocity ($v_{x}$(0), $v_{y}$(0), $v_{z}$(0)), and by solving Equation (A10), we obtain the following:

(A11) $$x\left(t\right)=x\left(0\right)-\frac{v_{z}\left(0\right)}{\omega }\left(1-cos\left(\omega t\right)\right)+\frac{v_{x}+E/B}{\omega }sin\left(\omega t\right)-\frac{E}{B}t$$

(A12) $$y\left(t\right)=y\left(0\right)+v_{y}\left(0\right)t$$

(A13) $$z\left(t\right)=z\left(0\right)+\frac{v_{x}\left(0\right)+E/B}{\omega }\left(1-cos\left(\omega t\right)\right)+\frac{v_{z}\left(0\right)}{\omega }sin\left(\omega t\right).$$

Here, ω=qB/m. Then, the transport time *T* required for the electrons to move from the conversion foil to the surface of the MCP detector is as follows:

$T=\frac{2\pi }{\omega }=\frac{2\pi m}{qB}$.

The maximum position of the SE in the z-axis with respect to the MCP surface is as follows:

(A14) $$z\left(T/2\right)=z\left(0\right)+\frac{v_{x}\left(0\right)+E/B}{\omega }\left(1-cos\left(\omega \frac{\pi }{\omega }\right)\right)=z\left(0\right)+2\frac{v_{x}\left(0\right)+E/B}{\omega }$$

Since the initial energy of the electron is very small, ignoring the influence of $v_{x}\left(0\right)$, z(T/2) can be approximated as follows:

(A15) $$z\left(T/2\right)=z\left(0\right)+\frac{E}{B\omega }\left(1-cos\left(\omega \frac{\pi }{\omega }\right)\right)=z\left(0\right)+\frac{2E}{B\omega }=z\left(0\right)+\frac{2mE}{qB^{2}}$$

The maximum height h of the SE motion in the z-axis direction with respect to the MCP surface is as follows:

(A16) $$h=z\left(T/2\right)-z\left(0\right)=\frac{2E}{B\omega }=\frac{2mE}{qB^{2}}$$

The distance D of the SE from the center of the conversion film in the x-direction to the center of the surface of the MCP detector is as follows:

(A17) $$D=x\left(T\right)-x\left(0\right)=\frac{E}{B}\frac{2\pi }{\omega }=\frac{2\pi mE}{qB^{2}}$$

The distance $Y_{T}$ from the center of the conversion film to the center of the surface of the MCP detector in the y-direction for a SE with an initial velocity of $v_{y}\left(0\right)$ in the y-direction is as follows:

(A18) $$Y_{T}=y\left(T\right)-y\left(0\right)=v_{y}\left(0\right)\frac{2\pi }{\omega }=v_{y}\left(0\right)\frac{2\pi m}{qB}$$

Normally, the initial energy of the SE is about a few eV, and the corresponding initial velocity v(0) is about ∼10^8^ cm/s. From the expression A17 of D, it can be seen that SEs are transported to the MCP surface from the emission start point along the x-direction at a fixed transport distance. The position along the y-direction depends on the magnitude of the initial velocity $v_{y}\left(0\right)$ (∼several mm). Therefore, this detector can be used to measure the precise position of the heavy ions in the transverse direction (x) when bombarding the conversion film, and the position of the SEs focused to the surface of the MCP in the x-direction does not depend on the initial velocity of the SEs. A simulation of the SE trajectory of ions from three points to the MCP is shown in Figure 1e.

Since the center of the conversion film and the MCP detector surface is fixed, the electron transport time is related to the ratio E/B of the magnetic field to the electric field strength. The velocity to the MCP is expressed by v=Dω, and the wobble of the electron transport time can be estimated as follows:

(A19) $$\delta T=\frac{v(0)}{\omega (E/B)}=\frac{m}{q}\frac{v(0)}{E}=2\pi \left(\frac{m}{q}\right)\frac{v(0)}{B^{2}D}$$

Usually, the structure is designed so that δT∼20 ps. In order to make the MCP detector have a high detection efficiency, there must be enough energy before the SE is incident on the MCP surface. Usually, there is a displacement difference between the surface of the MCP and the surface of the conversion film (carbon film).

#### Appendix A.2.1. Electrostatic and Magnetic Fields Parallelly Arranged MCP Detector

The electromagnetic field of the MCP detector shown in Figure 2b is arranged in parallel with the magnetic field, and this type of detector is commonly used for Bρ-TOF mass measurement experiments in NSCL/MSU. The position at a dispersive focal plane of the fragment separator is measured to obtain the momentum dispersion of the ions. High potential is applied to the conversion foil and MCP to generate an electric field.

The principle of the SE transport motion in the MCP detector with parallel electromagnetic fields is also shown in Figure 2b. A simulation of the SE transport motion from three points on the foil is shown in Figure 1d. The initial emission angle of the SE emitted from the conversion foil is $\theta_{i}$ (relative to the z-direction, see Figure 2b), the initial energy is $E_{0}$, the velocity is *v*, and the reverse of the electric field is $\vec{E}$. From the region with an initial magnetic field strength $B_{i}$, the SE undergoes a spiral motion to a final region with a magnetic field strength $B_{f}$; the angular frequency of the spiral motion is as follows: $\omega_{i}=eB/m$, where *e* is the charge and *m* the mass of an electron. The orbital radius of the spirally moving electron is as follows: $r_{i}=vsin\theta_{i}/\omega_{i}$. The orbital angular momentum of the electron is as follows: $l_{i}=\frac{m^{2}v^{2}sin^{2}\theta_{i}}{e\omega_{i}}$.

In this arrangement of the electromagnetic fields, the motion of electrons is an adiabatic process and angular momentum is a conserved quantity (adiabatic invariance):

(A20) $$\frac{sin\theta_{i}}{sin\theta_{i}}={\left(\frac{B_{i}}{B_{f}}\right)}^{1/2}$$

Under adiabatic invariance, the total velocity *v* of the electron is constant, and the longitudinal component of the velocity can be expressed as follows: $v_{f}=v{\left[1-\left(B_{f}/B_{i}\right){sin}^{2}\theta_{i}\right]}^{1/2}$.

It can be seen that the transverse energy of the electron motion is converted to the longitudinal direction, and the smaller the $B_{f}$/$B_{i}$, the smaller the transverse motion speed of the electron. We obtain the following:

(A21) $$\frac{r_{f}}{r_{i}}={\left(B_{i}/B_{f}\right)}^{1/2}$$

It can be seen that the offset of the electron motion to the surface of the MCP relative to the z-axis is proportional to M, which is the linear amplification factor of the transverse circular motion of the electron around the center:

(A22) $$M={\left(B_{i}/B_{f}\right)}^{1/2}$$

Equation (A20) and Equation (A22) hold only when the adiabatic limit condition (relative to the total field strength, the change in the field strength acting on the electrons is negligible) is satisfied. The varying field strength at this adiabatic limit is often referred to as the adiabatic parameter χ. The offset of electrons moving in a magnetic field with a longitudinal angle $\theta_{z}$ relative to the z-axis is $\Delta z_{ptich}$: $\Delta z_{ptich}=\frac{2\pi mv_{z}}{\omega }=\frac{2\pi mvcos\theta_{z}}{eB_{z}}$.

The change in field strength along the z-direction can be expressed as follows: $\Delta B_{z}=\frac{dB_{z}}{dz}\Delta z_{ptich}$, and χ can be expressed as follows:

(A23) $$\chi =\lim_{\theta_{z}\to 0}|\frac{\Delta B_{z}}{B_{z}}|=\frac{2\pi mv}{eB_{z}}\left|\frac{dB_{z}}{dz}\right|$$

where $\frac{dB_{z}}{dz}$ is the rate of the change in the magnetic field along the z-direction, and in order to meet the adiabatic condition, χ < 1 should be as close to 0 as possible.

## References

[1] Lunney D., Pearson J.M., Thibault C. (2003). Recent trends in the determination of nuclear masses. Rev. Mod. Phys..

[2] Blaum K. (2006). High-accuracy mass spectrometry with stored ions. Phys. Rev..

[3] Bianchi L., Fernandez B., Gastebois J., Gillibert A., Mittig W., Barrette J. (1989). SPEG: An energy loss spectrometer for GANIL. Nucl. Instr. Meth. Phys. Res. A.

[4] Wouters J.M., Vieira D.J., Wollnik H., Enge H.A., Kowalski S., Brown K.L. (1985). Optical design of the tofi (time-of-flight isochronous) spectrometer for mass measurements of exotic nuclei. Nucl. Instr. Meth. Phys. Res. A.

[5] Meisel Z., George S. (2013). Time-of-flight mass spectrometry of very exotic systems. Int. J. Mass Spectrom..

[6] Bollen G., Kluge H.-J., Otto T., Savard G., Stolzenberg H. (1992). Ramsey technique applied in a Penning trap mass spectrometer. Nucl. Instrum. Methods B.

[7] Lunney D., on behalf of the ISOLTRAP Collaboration (2017). Extending and refining the nuclear mass surface with ISOLTRAP. J. Phys. G Nucl. Part. Phys..

[8] Eronen T., Kolhinen V.S., Elomaa V.V., Gorelov D., Hager U., Hakala J., Jokinen A., Kankainen A., Karvonen P., Kopecky S. (2012). JYFLTRAP: A Penning trap for precision mass spectroscopy and isobaric purification. Eur. Phys. J. A.

[9] Rahaman S., Block M., Ackermann D., Beck D., Chaudhuri A., Eliseev S., Geissel H., Habs D., Herfurth F., Heßberger F.P. (2006). On-line commissioning of SHIPTRAP. Int. J. Mass Spec..

[10] Grund J., Asai M., Blaum K., Block M., Chenmarev S., Düllmann C.E., Eberhardt K., Lohse S., Nagame Y., Nagy S. (2020). First online operation of TRIGA-TRAP. Nucl. Instrum. Methods Phys. Res. A.

[11] Savard G., Wang J.C., Sharma K.S., Sharma H., Clark J.A., Boudreau C., Buchinger F., Crawford J.E., Greene J.P., Gulick S. (2006). Studies of neutron-rich isotopes with the CPT mass spectrometer and the CARIBU project. Int. J. Mass Spectrom..

[12] Ringle R., Schury P., Sun T., Bollen G., Davies D., Huikari J., Kwan E., Morrissey D.J., Prinke A., Savory J. (2006). Precision mass measurements with LEBIT at MSU. Int. J. Mass Spectrom..

[13] Bergström I., Carlberg C., Fritioff T., Douysset G., Schönfelder J., Schuch R. (2002). SMILETRAPFA Penning trap facility for precision mass measurements using highly charged ions. Nucl. Instrum. Methods A.

[14] Bosch F., Litvinov Y.A., Stöhlker T. (2013). Nuclear physics with unstable ions at storage rings. Prog. Part. Nucl. Phys..

[15] Hausmann M., Attallah F., Beckert K., Bosch F., Dolinskiy A., Eickhoff H., Falch M., Franczak B., Franzke B., Geissel H. (2000). First isochronous mass spectrometry at the experimental storage ring ESR. Nucl. Instrum. Methods A.

[16] Geissel H., Knöbel R., Litvinov Y.A., Sun B., Beckert K., Bélier P., Bosch F., Brandau C., Kozhuharov C., Kurcewicz J. (2006). A new experimental approach for isochronous mass measurements of short-lived exotic nuclei with the FRS-ESR facility. Hyperfine Interact..

[17] Sun M.Z., Zhou X.H., Wang M., Zhang Y., Litvinov Y.A. (2018). Precision mass measurements of short-lived nuclides at HIRFL-CSR in Lanzhou. Front. Phys..

[18] Wolf R.N., Wienholtz F., Atanasov D., Beck D., Blaum K., Borgmann C., Herfurth F., Kowalska M., Kreim S., Litvinov Y.A. (2013). ISOLTRAP’s multi-reflection time-of-flight mass separator/spectrometer. Int. J. Mass Spectrom.

[19] Plaß W.R., Dickel T., Purushothaman S., Dendooven P., Geissel H., Ebert J., Yavor M.I. (2013). The FRS Ion Catcher—A facility for high-precision experiments with stopped projectile and fission fragments. Nucl. Instrum. Methods Phys. Res. B.

[20] Rosenbusch M., Wada M., Chen S., Takamine A., Iimura S., Hou D., Xian W., Yan S., Schury P., Hirayama Y. (2023). The new MRTOF mass spectrograph following the ZeroDegree spectrometer at RIKEN’s RIBF facility. Nucl. Instrum. Methods Phys. Res. A.

[21] Kuzminchuk-Feuerstein N., Fabian B., Diwisch M., Plaß W.R., Geissel H., San Andrés S.A., Dickel T., Knöbel R., Scheidenberger C., Sun B. (2016). Efficiency and rate capability studies of the time-of-flight detector for isochronous mass measurements of stored short-lived nuclei with the FRS-ESR facility. Nucl. Instrum. Meth. Phys. Res. A.

[22] Diwisch M., Plaß W.R., Geissel H., Knöbel R., Kuzminchuk-Feuerstein N., San Andrés S.A., Dickel T., Scheidenberger C., Weick H. (2015). Design of a new time-of-flight detector for isochronous mass spectrometry in the collector ring at FAIR. Phys. Scr..

[23] https://www.gsi.de/.

[24] Mei B., Tu X., Wang M., Xu H., Mao R., Hu Z., Ma X., Yuan Y., Zhang X., Geng P. (2010). A high performance Time-of-Flight detector applied to isochronous mass measurement at CSRe. Nucl. Instrum. Methods Phys. Res. Sect. A.

[25] Zhang W., Tu X.L., Wang M., Zhang Y.H., Xu H.S., Litvinov Y.A., Blaum K., Chen R.J., Chen X.C., Fu C.Y. (2014). Time-of-flight detectors with improved timing performance for isochronous mass measurements at the CSRe. Nucl. Instrum. Methods Phys. Res. Sect. A.

[26] http://english.imp.cas.cn/.

[27] Suzuki S., Ozawa A., Kamioka D., Abe Y., Amano M., Arakawa H., Ge Z., Hiraishi K., Ichikawa Y., Inomata K. (2020). Efficiency and timing performance of time-of-flight detector utilizing thin foils and crossed static electric and magnetic fields for mass measurements with Rare-RI Ring facility. Nucl. Instrum. Methods A.

[28] Suzuki1 S., Ozawa A., Moriguchi T., Amano M., Kamioka D., Ichikawa Y., Tajiri Y., Hiraishi K., Matsumoto T., Nagae D. (2017). In INPC2016: Performance of Time-Of-Flight Detector and Demonstration of Completely New Position Detector for Mass Measurements with the Rare-RI Ring. Proc. Sci..

[29] Nagae D., Abe Y., Okada S., Ozawa A., Yamaguchi T., Suzuki H., Moriguchi T., Ishibashi Y., Fukuoka S., Nishikiori R. (2013). Time-of-flight detector applied to mass measurements in Rare-RI Ring. Nucl. Instrum. Methods Phys. Res. Sect. B.

[30] Ge Z., Naimi S., Nagae D., Abe Y., Omika S., Uesaka T., Suzaki F., Li H., Yamaguchi Y., Wakasugi M. (2021). Development of Mirror-type MCP Detectors for Mass Measurements at the Rare-RI Ring. JPS Conf. Proc..

[31] Nagae D., Abe Y., Okada S., Omika S., Wakayama K., Hosoi S., Suzuki S., Moriguchi T., Amano M., Kamioka D. (2021). Development and operation of an electrostatic time-of-flight detector for the Rare RI storage Ring. Nucl. Instrum. Methods Phys. Res. Sect. A.

[32] http://www.riken.jp/en/.

[33] Matoš M., Estradé A., Schatz H., Bazin D., Famiano M., Gade A., George S., Lynch W.G., Meisel Z., Portillo M. (2012). Time-of-flight mass measurements of exotic nuclei. Nucl. Instr. Meth. Phys. Res. A.

[34] Ge Z., Naimi S., Nagae D., Abe Y., Uesaka T., Yamaguchi T., Suzaki F., Yamaguchi Y., Wakasugi M., Omika S. (2017). Simulation and design of a large area position sensitive TOF MCP detector at the Rare RI Ring. RIKEN Accel. Prog. Rep..

[35] Ge Z., Uesaka T., Naimi S., Nagae D., Abe Y., Omika S., Suzaki F., Yamaguchi T., Yamaguchi Y., Wakusugi M. (2019). Scheme of high-resolution identification and selection of secondary ions for mass measurements with the Rare-RI Ring. Hyperfine Interact..

[36] Ge Z., Li H.F., Uesaka T., Naimi S., Nagae D., Abe Y., Omika S., Suzaki F., Yamaguchi Y., Wakasugi M. (2021). Development of a large-area timing and position-sensitive foil-MCP detector for mass measurements at the Rare-RI Ring in RIKEN. arXiv.

[37] Geissel H., Franczak B., Haettner E., Ge Z., Dickel T., Kuzminchuk-Feuerstein N., Litvinov S., Litvinov Y.A., Patyk Z., Plaß W.R. (2023). Novel isochronous features for FRS-ESR experiments with stored exotic projectile fragments. Nucl. Instr. Meth. Phys. Res. B.

[38] Liu J.H., Ge Z., Wang Q., Wang G., Sheng L., Ge W.W., Xu X., Shuai P., Zeng Q., Wu B. (2019). Electrostatic-lenses position-sensitive TOF MCP detector for beam diagnostics and new scheme for mass measurements at HIAF. Nucl. Sci. Tech..

[39] Rehm K.E., Wolfs F.L.H. (1988). A focal plane detector for reactions with medium weight projectiles. Nucl. Instr. Meth. A.

[40] Breskin A., Chechik R., Fraenkel Z., Jacobs P., Tserruya I., Zwang N. (1984). Low-pressure multisteo detector for very low energy heavy ions. Nucl. Instr. Meth..

[41] Kumagai H., Ozawa A., Fukuda N., Sümmerer K., Tanihata I. (2001). Delay-line PPAC for high-energy light ions. Nucl. Instrum. Meth. A.

[42] Kumagai H., Ohnishi T., Fukuda N., Takeda H., Kameda D., Inabe N., Yoshida K., Kubo T. (2013). Development of Parallel Plate Avalanche Counter (PPAC) for BigRIPS fragment separator. Nucl. Instr. Meth. Phys. Res. B.

[43] Charpak G., Sauli F. (1979). Multiwire Proportional Chambers and Drift Chambers. Nucl. Instrum. Methods.

[44] Hlinka V., Ivanov M., Janik R., Sitar B., Strmen P., Szarka I., Baumann T., Geissel H., Schwab W. (1998). Time projection chambers for tracking and identification of radioactive beams. Nucl. Instrum. Methods Phys. Res. Sect. A.

[45] Goldstone P.D., Malmin R.E., Hopkins F., Paul P. (1974). Development of thin-film plastic scintillator detectors for use in in-beam fission studies. Nucl. Instr. Meth..

[46] Gabor G., Schimmerling W., Greiner D., Bleser F., Lindstrom P. (1975). High resolution spectrometry for relativistic heavy ions. Nucl. Instr. Meth..

[47] Pollacco E.C., Jacmart J.C., Blumenfeld Y., Chomaz P., Frascaria N., Garron J.P., Roynette J.C. (1984). A compact gridless channel plate detector for time-of-flight measurements. Nucl. Instr. Meth..

[48] Culhane J.L. (1991). Position sensitive detectors in X-ray astronomy. Nucl. Instr. Meth..

[49] Wiza J.L. (1979). Microchannel Plate Detectors. Nucl. Instr. Meth. Phys. Res. Sect..

[50] Starzecki W., Stefanini A.M., Lunardi S., Signorini C. (1982). A compact time-zero detector for mass identification of heavy ions. Nucl. Instr. Meth..

[51] Kosev K., Nankov N., Friedrich M., Grosse E., Hartmann A., Heidel K., Junghans A.R., Schilling K.D., Schwengner R., Sobiella M. (2008). A high-resolution time-of-flight spectrometer with tracking capabilities for fission fragments and beams of exotic nuclei. Nucl. Instr. Meth. Phys. Res. A.

[52] Arnold C.W., Tovesson F., Meierbachtol K., Bredeweg T., Jandel M., Jorgenson H.J., Laptev A., Rusev G., Shields D.W., White M. (2014). Development of position-sensitive time-of-flight spectrometer for fission fragment research. Nucl. Instr. Meth. Phys. Res. A.

[53] Morimoto K., Mayama K., Kaji D., Tokanai F. (2013). Development of a large area TOF detector for super-heavy element research. RIKEN Accel. Prog. Rep..

[54] Ishizawa S., Morimoto K., Kaji D., Tanaka T., Tokanai F. (2020). Improvement of the detection efficiency of a time-of-flight detector for superheavy element search. Nucl. Instr. Meth. Phys. Res. A.

[55] Laitinen M., Rossi M., Julin J., Sajavaara T. (2014). Secondary electron flight times and tracks in the carbon foil time pick-up detector. Nucl. Instrum. Methods Phys. Res. B.

[56] Steinbach T.K., Vadas J., Schmidt J., Haycraft C., Hudan S., Desouza R.T., Baby L.T., Kuvin S.A., Wiedenhöver I., Umar A.S. (2014). Sub-barrier enhancement of fusion as compared to a microscopic method in ^18^O + ^12^C. Phys. Rev. C.

[57] Singh V., Vadas J., Steinbach T.K., Wiggins B.B., Hudan S., de Souza R.T., Lin Z., Horowitz C.J., Baby L.T., Kuvin S.A. (2017). Fusion enhancement at near and sub-barrier energies in ^19^O + ^12^C. Phys. Lett. B.

[58] Eliseev S., Blaum K., Block M., Dörr A., Droese C., Eronen T., Goncharov M., Höcker M., Ketter J., Ramirez E.M. (2014). A phase-imaging technique for cyclotron-frequency measurements. Appl. Phys. B.

[59] Eliseev S., Blaum K., Block M., Droese C., Goncharov M., Minaya Ramirez E., Nesterenko D.A., Novikov Y.N., Schweikhard L. (2013). Phase-imaging ion-cyclotron-resonance measurements for short-lived nuclides. Phys. Rev. Lett..

[60] Nesterenko D.A., Eronen T., Kankainen A., Canete L., Jokinen A., Moore I.D., Penttilä H., Rinta-Antila S., de Roubin A., Vilén M. (2018). Phase-Imaging Ion-Cyclotron-Resonance technique at the JYFLTRAP double Penning trap mass spectrometer. Eur. Phys. J. A.

[61] Nesterenko D.A., Eronen T., Ge Z., Kankainen A., Vilen M. (2021). Study of radial motion phase advance during motion excitations in a Penning trap and accuracy of JYFLTRAP mass spectrometer. Eur. Phys. J. A.

[62] Niwase T., Wada M., Schury P., Haba H., Ishizawa S., Ito Y., Kaji D., Kimura S., Miyatake H., Morimoto K. (2020). Development of an ‘‘alpha-TOF’’ detector for correlated measurement of atomic masses and decay properties. Nucl. Instrum. Meth. Phys. Res. A.

[63] Niwase T., Xian W., Wada M., Rosenbusch M., Chen S., Takamine A., Liu J., Iimura S., Hou D., Yan S. (2023). Development of a beta-TOF detector: An enhancement of the alpha-TOF detector for use with beta-decaying nuclides. Prog. Theor. Exp. Phys..

[64] Tremsin A.S., Vallerga J.V., Siegmund O.H.W. (2020). Overview of spatial and timing resolution of event counting detectors with Microchannel Plates. Instrum. Methods Phys. Res. A.

[65] Fraser G.W. (1984). X- and gamma-ray imaging using microchannel plates. Nucl. Instrum. Meth. Phys. Res..

[66] Sobottka S.E., Williams M.B. (1988). Delay line readout of microchannel plates. IEEE Trans. Nucl. Sci..

[67] Hong R., Leredde A., Bagdasarova Y., Fléchard X., Garcia A., Müller P., Knecht A., Liénard E., Kossin M., Sternberg M.G. (2016). High accuracy position response calibration method for a micro-channel plate ion detector, Nucl. Instrum. Meth. A.

[68] Siegmund O., Tremsin A., Vallerga J., McPhate J. (2009). Microchannel plate cross-strip detectors with high spatial and temporal resolution. Nucl. Instrum. Meth. A.

[69] de Souza R.T., Gosser Z.Q., Hudan S. (2012). Using induced signals to sense position from a microchannel plate detector. Rev. Sci. Instrum..

[70] de Souza R.T., Wiggins B.B., Siwal D. Sensing an electron cloud emanating from a microchannel plate stack. Proceedings of the 2015 IEEE Nuclear Science Symposium and Medical Imaging Conference (NSS/MIC).

[71] Lampton M., Carlson C.W. (1979). Low-distortion resistive anodes for two-dimensional position-sensitive MCP systems. Rev. Sci. Instrum..

[72] de Souza R.T., Wiggins B.B., Siwal D. (2015). Sensing an electron cloud emanating from a microchannel plate stack. Rev. Sci. Instrum..

[73] Siwal D., Wiggins B.B., de Souza R.T. (2015). Using pulse shape analysis to improve the position resolution of a resistive anode microchannel plate detector. Nucl. Instrum. Meth. A.

[74] Tremsin A.S., McPhate J.B., Vallerga J.V., Siegmund O.H.W., Feller W.B., Lehmann E., Butler L.G., Dawson M. (2011). High-resolution neutron microtomography with noiseless neutron counting detector. Nucl. Instrum. Meth. A.

[75] James A.N., Morrison T.P., Ying K.L., Connell K.A., Price H.G., Simpson J. (1988). Microsecond mass separation of heavy compound nucleus residues using the Daresbury recoil separator. Nucl. Instr. Meth. A.

[76] Shapira D., Lewis T.A., Hulett L.D., Ciao Z. (2000). Factors affecting the performance of detectors that use secondary electron emission from a thin foil to determine ion impact position. Nucl. Instr. Meth. A.

[77] Odland O.H., Mittig W., Lepine-Szily A., Fremont G., Chartrier M., MacCormick M., Casandjian J.M. (1996). A fast position sensitive microchannel plate detector for ray-tracing of charged particles. Nucl. Instr. Meth. A.

[78] Rogers A.M., Sanetullaev A., Lynch W.G., Tsang M.B., Lee J., Bazin D., Coupl D., Henzl V., Henzlova D., Kilburn M. (2015). Tracking rare-isotope beams with microchannel plates. Nucl. Instrum. Methods Phys. Res. Sect. A.

[79] Bowman J.D., Heffner R.H. (1978). A novel zero time detector for heavy ion spectroscopy. Nucl. Instrum. Meth. Phys. Res..

[80] Kraus R.H., Vieira D.J., Wollnik H., Wouters J.M. (1988). Large-area fast-timing detectors developed for the TOFI spectrometer. Nucl. Instrum. Meth. A.

[81] Odenweller T., Noll H., Sapotta K., Renfordt R.E., Bass R. (1982). A gridless position sensitive time-zero detector for heavy ions. Nucl. Instrum. Meth. Phys. Res..

[82] Steinbach T.K., Rudolph M.J., Gosser Z.Q., Brown K., Floyd B., Hudan S., Desouza R.T., Liang J.F., Shapira D., Famiano M. (2014). Measuring the fusion cross-section of light nuclei with low-intensity beams. Nucl. Instrum. Meth. A.

[83] Wiggins B.B., Singh V., Vadas J., Huston J., Steinbach T.K., Hudan S., de Souza R.T. (2017). Development of a compact E×B microchannel plate detector for beam imaging. Nucl. Instrum. Methods Phys. Res. Sect. A.

[84] Ge Z. (2018). Time and Position-sensitive Foil MCP Detector for Mass Measurements at the Rare-RI Ring. Ph.D. Thesis.

[85] Dahl D.A. SIMION 3D. https://simion.com.

[86] Šaro Š, Janik R., Hofmann S., Folger H., Heßberger F.P., Ninov V., Schött H.J., Kabachenko A.P., Popeko A.G., Yeremin A.V. (1996). Large size foil-microchannel plate timing detectors. Nucl. Instrum. Methods Phys. Res. Sect. A.

[87] Rothard H., Kroneberger K., Clouvas A., Veje E., Lorenzen P., Keller N., Kemmler J., Meckbach W., Groeneveld K.O. (1990). Secondary electron yields from thin foils: A possible probe for the electronic stopping power of heavy ions. Phys. Rev. A.

[88] Shima K., Ishii S., Takahashi T., Sugai I. (2001). Optimum thickness of carbon stripper foils in tandem accelerator in view of transmission and lifetime. Nucl. Instr. Meth. A.

[89] Ma W., Liechtenstein V.K., Szerypo J., Jung D., Hilzx P., Hegelich B.M., Maier H.J., Schreiber J., Habs D. (2011). Preparation of selfsupporting diamond-like carbon nanofoils with thickness less than 5 nm for laser driven ion acceleration. Nucl. Instr. Meth. A.

[90] Liechtenstein V.K., Ivkova T.M., Olshanski E.D., Repnow R., Steier P., Kutschera W., Wallner A., von Hahn R. (2006). Preparation and investigation of ultra-thin diamond-like carbon (DLC) foils reinforced with collodion. Nucl. Instr. Meth. A.

[91] Hasselkamp D., Rothard H., Groeneveld K.O., Kemmler J., Varga P., Winter H. (2006). Particle Induced Electron Emission II.

[92] Drexler C.G., DuBois R.D. (1996). Energy- and angle-differential yields of electron emission from thin carbon foils after fast proton impact. Phys. Rev. A.

[93] Rothard H., Caraby C., Cassimi A., Gervais B., Grandin J.P., Jardin P., Jung M., Billebaud A., Chevallier M., Groeneveld K.O. (1995). Target-thickness-dependent electron emission from carbon foils bombarded with swift highly charged heavy ions. Phys. Rev. A.

[94] Sternglass E.J. (1957). Theory of secondary electron emission by high speed ions. Phys. Rev..

[95] Koschar P., Kroneberger K., Clouvas A., Burkhard M., Meckbach W., Heil O., Kemmler J., Rothard H., Groeneveld K.O., Schramm R. (1989). Secondary-electron yield as a probe of preequilibrium stopping power of heavy ions colliding with solids. Phys. Rev. A.

[96] Hasselkamp D., Hippler S., Scharmann A. (1987). Ion-induced secondary electron spectra from clean metal surfaces. Nucl. Instr. Meth. Phys. Res. B.

[97] Rösler M., Brauer W., Devooght J., Dehaes J., Dubus A., Cailler M., Ganachaud J.-P. (2006). Particle Induced Electron Emission I.

[98] Jung M., Rothard H., Gervais B., Grandin J.P., Clouvas A., Wünsch R. (1996). Transport of electrons induced by highly charged Ni (74 MeV/u) and Cu (9.6 MeV/u) ions in carbon: A study of target-thickness-dependent electron yields. Phys. Rev. A.

[99] Clouvas A., Rothard H., Burkhard M., Kroneberger K., Biedermann C., Kemmler J., Groeneveld K.O., Kirsch R., Misaelides P., Katsanos A. (1989). Secondary electron emission from thin foils under fast-ion bombardment. Phys. Rev. B.

[100] Galanti M., Gott R., Renaud J.F. (1971). A High Resolution, High Sensitivity Channel Plate Image Intensifier for Use in Particle Spectrographs. Rev. Sci. Instrum..

[101] Agranovich V.M., Daukeev D.K., Konobeev Y.V., Lebedev S.Y. (1970). Study of the energy spectrum of secondary electrons arising from passage of *α* particles and fission fragmenst trough thin foils. Soviet. Phys. JETP.

[102] Shapira D., Lewis T.A., Hulett L.D. (2000). A fast and accurate position-sensitive timing detector based on secondary electron emission. Nucl. Instrum. Meth. A.

[103] Ge Z. (2019). Design and test of high resolution beam-line and mass measurements of N=Z nuclei. Ph.D. Thesis.

[104] Ozawa A., Uesaka T., Wakasugi M. (2012). The Rare-RI Ring Collaboration, The rare-RI ring. Prog. Theor. Exp. Phy..

[105] RoentDek GmbH. http://www.roentdek.com.

[106] https://www.bmtdynamics.org/cosy/.

[107] https://web-docs.gsi.de/~weick/mocadi/.

[108] http://www.kaizuworks.co.jp.

[109] http://www.hamamatsu.com/jp/en/index.html.

[110] Hirao Y.O.H.Y., Ogawa H., Yamada S., Sato Y., Yamada T., Sato K., Itano A., Kanazawa M., Noda K., Kawachi K. (1992). Heavy ion synchrotron for medical use—HIMAC project at NIRS-Japan. Nucl. Phys. A.

[111] Kanazawa M., Kitagawa A., Kouda S., Nishio T., Torikoshi M., Noda K., Murakami T., Sato S., Suda M., Tomitani T. (2004). Present status of secondary beam courses in HIMAC. Nucl. Phys. A.

[112] Yano Y. (2007). The RIKEN RI Beam Factory Project: A status report. Nucl. Instr. Meth. Phys. Res. B.

[113] Yang J.C., Xia J.W., Xiao G.Q., Xu H.S., Zhao H.W., Zhou X.H., Ma X.W., He Y., Ma L.Z., Gao D.Q. (2013). High Intensity heavy ion Accelerator Facility (HIAF) in China. Nucl. Instr. Meth. Phys. Res. A.

[114] Chen X., Shen L.N., Yang J.C., Zhang X., Zhang J., Mao L., Wu B., Zhao H., Ruan S., Wang J. (2017). Separation performance research of superconducting fragment separator. High Power Laser Part. Beams.

[115] Geissel H., Litvinov Y.A. (2005). Precision experiments with relativistic exotic nuclei at GSI. J. Phys. G Nucl. Part. Phys..

[116] Xing Y.M., Wang M., Zhang Y.H., Shuai P., Xu X., Chen R.J., Yan X.L., Tu X.L., Zhang W., Fu C.Y. (2015). First isochronous mass measurements with two time-of-flight detectors at CSRe. Phys. Scrip..

[117] Walker P.M. (2005). Technical Proposal for the ILIMA Project. https://nustar-wiki.gsi.de/foswiki/pub/ILIMA/TechnicalProposal/ILIMA_TP_20051215.pdf.

[118] Ge Z. To Be Submitted to. https://arxiv.org.

